# Phylogenetic divergences in brown rot fungal pathogens of *Monilinia* species from a worldwide collection: inferences based on the nuclear versus mitochondrial genes

**DOI:** 10.1186/s12862-022-02079-6

**Published:** 2022-10-21

**Authors:** Ece Silan, Hilal Ozkilinc

**Affiliations:** 1 School of Graduate Studies, MSc Program in Biomolecular Sciences, Çanakkale Onsekiz Mart University, Çanakkale, Turkey; 2 Dept. of Molecular Biology and Genetics, Faculty of Science, Çanakkale Onsekiz Mart University, Çanakkale, Turkey

**Keywords:** Phylogenetics, *Monilinia*, Mitochondrial gene evolution, Nuclear gene evolution

## Abstract

**Background:**

Phylogenetic analyses for plant pathogenic fungi explore many questions on diversities, relationships, origins, and divergences of populations from different sources such as species, host, and geography. This information is highly valuable, especially from a large global sampling, to understand the evolutionary paths of the pathogens worldwide. *Monilinia fructicola* and *M. laxa* are two important fungal pathogens of stone fruits that cause the widespread disease commonly known as brown rot. Three nuclear genes (*Calmodulin*, *SDHA*, *TEF1α*) and three mitochondrial genes (*Cytochrome_b*, *NAD2*, and *NAD5*) of the two pathogen species from a worldwide collection including five different countries from four different continents were studied in this work.

**Results:**

Both Maximum Likelihood and Bayesian approaches were applied to the data sets, and in addition, Maximum Parsimony based approaches were used for the regions having indel polymorphisms. *Calmodulin*, *SDHA*, *NAD2,* and *NAD5* regions were found phylogenetically informative and utilized for phylogenetics of *Monilinia* species for the first time. Each gene region presented a set of haplotypes except *Cytochrome_b*, which was monomorphic. According to this large collection of two *Monilinia* species around the world, *M. fructicola* showed more diversity than *M. laxa*, a result that should be carefully considered, as *M. fructicola* is known to be a quarantine pathogen. Moreover, the other two mitochondrial genes (*NAD2* and *NAD5*) did not have any substitution type mutations but presented an intron indel polymorphism indicating the contribution of introns as well as mobile introns to the fungal diversity and evolution. Based on the concatenated gene sets, nuclear DNA carries higher mutations and uncovers more phylogenetic clusters in comparison to the mitochondrial DNA-based data for these fungal species.

**Conclusions:**

This study provides the most comprehensive knowledge on the phylogenetics of both nuclear and mitochondrial genes of two prominent brown rot pathogens, *M. fructicola* and *M. laxa*. Based on the regions used in this study, the nuclear genes resolved phylogenetic branching better than the mitochondrial genes and discovered new phylogenetic lineages for these species.

**Supplementary Information:**

The online version contains supplementary material available at 10.1186/s12862-022-02079-6.

## Background

Understanding the origin and diversification of living organisms is essential for discovering and predicting their evolutionary trajectories. Especially for pathogenic fungi, gathering information on the origin and roots of the lineages, revealing newly emerging species, and assessing relatedness of pathogen populations across the world are helpful for designing and implementing disease management strategies.

Brown rot pathogens of *c* species cause severe economic losses on stone and pome fruits worldwide [1, 2]. The disease is prominent on fruits, but the symptoms can also be observed on leaves and blossoms, flowers, and twigs [1–5]. Among the three most known species, *M. fructicola* and *M. laxa* infect mainly stone fruits whereas *M. fructigena* is a pathogen of pome fruits [2]. While these species have been known since the early 1900s, new pathogenic species of *Monilinia* have been detected in the last twenty years as a result of molecular phylogenetic studies e.g., *M. polystroma* [6–8], reported from both Asia and Europe; *M. mumecola* [9, 10] and *M. yunnanensis* [11] from Asia. While newly discovered *Monilinia* species are reported specifically from localized regions, *M. fructicola* and *M. laxa* are widespread and have been reported from every continent that can infect, including Africa [12], Asia [11, 13], Europe, North America [14] and South America [15], and even in a closed environment such as Australia [16].

The first phylogenetic studies on genus *Monilinia* were mostly focused on the characterization and identification of the species. In 1997, Fulton and Brown designed a marker from a group-I intron in the SSU rDNA gene that aimed to distinguish *M. fructicola* from *M. laxa* and *M. fructigena* [17]. In the same year, coding and noncoding rDNA sequences were used in a phylogenetic study including 17 *Monilinia* species [18]. In 2004, Cote designed three species-specific forward primers (MO368-8R, MO368-10R, and Laxa-R2) and a common reverse primer (MO268-5) that yielded different-sized PCR products and help discerning among these species [19]. Afterwards, other studies emerged, which analysed new sets of genes that could be used in determining genetic variability within and among species. A set of gene regions studied in *Botrytis cinerea*, a plant pathogen that has over 1000 hosts [20] and is one of the most closely related species to the *Monilinia* genus, were tested in brown rot pathogens. In a study in 2011, *glyceraldehyde-3-phosphate dehydrogenase* (*G3PDH*), *beta-tubulin* (*tub2*) and mitochondrial *cytochrome b* (*Cytb*) markers were designed for characterization and phylogenetic inferences of *Monilinia* species from peach hosts in China [11]. Maximum parsimony and neighbour-joining trees based on a concatenated dataset including these three markers plus the ITS region identified a new diverse group closely related to *M. fructigena* that was named *M. yunnanensis* [11]. However, while these different genomic regions were phylogenetically informative enough to distinguish among species, they did not resolve distinct lineages within species [11]. In another study, phylogenetic trees of *M. fructicola* and *M. laxa* samples mainly from Brazil were inferred based on partial sequences of *TEF1*a, ITS, and *RBP2* regions. The phylogenetic analyses found that *TEF1*a was the most informative marker [15]. Recently, regions *hsp60*, *acp1,* and *pac1* were also characterized and used in a phylogenetic study for *M. fructigena, M. polystroma, M. laxa,* and *M. fructicola* from Poland [21]. All these studies were able to distinguish the different *Monilinia* species in the phylogenetic trees, but had limited resolution at the intraspecific level. Up to now, phylogenetic studies on *Monilinia* species have included only a couple of nuclear regions and one mitochondrial region as well as a limited number of samples from different countries [11, 15, 21]. In this regard, new studies with more genes and samples on the phylogenetic relationships of *Monilinia* species distributed worldwide would be very helpful to understand the evolutionary trajectory of the pathogen, as well as the disease.

Furthermore, nuclear and mitochondrial genome evolution rates differ as shown in numerous studies [22–24]. In animals, mutation rate is higher in the mitochondrial genome than in the nuclear genome [23, 25]. On the contrary, for many fungal species, low mutation rates in mitochondrial genomes compared to the nuclear genomes have been reported based on RFLP-based markers [26, 27], multilocus sequences [28, 29] and whole-genome sequences [30]. Yet, for other fungi, higher mutation rates in mitochondrial genomes than nuclear genomes were reported [22, 31]. Since both genomic sources are important to shape pathogen population structures and evolution, comparing/contrasting mitochondrial and nuclear gene phylogenies should be considered mandatory.

Understanding species diversification, on a taxonomically smaller but geographically larger scale and estimating evolution rates using both molecular and mitochondrial markers, will help to understand the evolutionary trajectories of the pathogens as well as their management. Considering the current limited available information on the phylogenetic relationships of *Monilinia* species, we aimed to study in depth genetic diversity and phylogenetics of the most prevalent *Monilinia* pathogen species (*M. fructicola* and *M. laxa*) from different countries and hosts by using sequence data from the selected nuclear and mitochondrial genes. This study helps to resolve the global molecular evolution of these two important plant pathogens by considering mutations from both nuclear and mitochondrial genes as well as by introducing new informative regions to be used in *Monilinia* phylogenetics.

## Results

### Sequences, haplotypes, and DNA polymorphisms

Sequences of each region were amplified and Sanger sequenced, and then manually trimmed to be used in alignments. Amplified regions of *Calmodulin* were around 310 bp and *SDHA* regions were around 800 bp for both species. *TEF1* region of *M. fructicola* isolates consisted of 440 bp sequences whereas *M. laxa* isolates were around 270 bp. For mitochondrial regions, amplified *Cytb* sequences of *M. fructicola* were around 600 bp whereas *M. laxa* sequences were 480 bp. *NAD5* sequences were around 670 bp long for *M. laxa* and *M. fructicola* isolates which carried the intron. *Monilinia fructicola* isolates without the mobile intron of *NAD5* gene had sequences of about 250 bp. *NAD2* region including the mobile intron was about 760 bp for the isolates of both species. The *NAD2* region of the rest of *M. fructicola* isolates resulted in about 570 bp. Every sequence was checked by using the NCBI/BLASTn tool. All regions except mitochondrial *NAD2* and *NAD5* rendered 100% query coverage with over 90% percent identities with *Monilinia* species deposited in GenBank. *NAD2* and *NAD5* hits aligned with already published *Monilinia* mt-genome sequences [32]. The new sequences of the nuclear DNA regions were uploaded in GenBank with accession numbers OP036604–OP036610 for *Calmodulin*, OP090593–OP090612 for *SDHA*, and OP090613–OP090640 for *TEF1*.

For phylogenetic analyses, haplotypes were determined and named for each region (Additional file 9: Table S1). Eleven haplotypes for the *SDHA* region (9 for *M. fructicola*, 2 for *M. laxa*), 7 haplotypes were *Calmodulin* (6 for *M. fructicola*, 1 for *M. laxa*), 16 haplotypes (10 *M. fructicola*, 6 *M. laxa*) for *TEF1a* were determined. The *Cytb* region did not show any diversity; therefore, a single haplotype was observed for each species. For *NAD5* and *NAD2* regions, only three haplotypes were determined, two for *M. fructicola* due to mobile intron presence/absence, and one for *M. laxa*.

The *SDHA* and *TEF1* nuclear gene regions in both species together with *NAD5* in *M. fructicola* presented the greatest number of polymorphic sites. *Calmodulin* had only four polymorphic sites in *M. fructicola* and was monomorphic in *M. laxa*. *Cytb* and *NAD2* in both species and *NAD5* in *M. laxa* were also monomorphic (Table 1). Based on the sequence data from both species, nucleotide diversities (π) were recorded as 0.057 for *TEF1* and 0.033 for *SDHA,* and 0.030 for *Calmodulin* (Table 1). Concatenated sequences for both nuDNA and mtDNA regions showed higher nucleotide diversity for *M. fructicola* species (π = 0.0047 and 0.01962, respectively) in comparison to the diversity of *M. laxa* (π = 0.00083 and 0.00037, respectively) (Table 2). Overall, the nuclear DNA regions were found to be more polymorphic than the mitochondrial regions (Table 2).

**Table 1:** Table of polymorphism statistics of each region

	*Calmodulin*	*SDHA*	*TEF1*	*NAD2*	*NAD5*	*CYTB*
*M. fructicola* & *M. laxa*						
Number of haplotypes	7	11	16	3	3	2
Polymorphic sites	23	62	60	105	7	30
Total number of mutations	23	63	61	105	7	30
Singleton variable sites	1	2	4	0	0	0
Parsimony informative sites	22	60	56	105	7	30
Nucleotide diversity, Pi	0.03066	0.033	0.0579	0.02529	0.0175	0.029
*M. laxa*						
Number of haplotypes	1	2	6	1	1	1
Polymorphic sites	0	1	4	0	0	0
Total number of mutations	0	1	4	0	0	0
Singleton variable sites	0	0	2	0	0	0
Parsimony informative sites	0	1	2	0	0	0
Nucleotide diversity, Pi	0	7E−04	0.0579	0	0	0
*M. fructicola*						
Number of haplotypes	6	9	10	2	2	1
Polymorphic sites	4	7	8	0	7	0
Total number of mutations	4	7	8	0	7	0
Singleton variable sites	2	3	3	0	0	0
Parsimony informative sites	2	4	5	0	7	0
Nucleotide diversity, Pi	0.00365	0.003	0.0057	0	0.0175	0
First part of the table consists of both *M. fructicola* and *M. laxa* isolates, whereas second and third parts show only *M. laxa* and *M. fructicola* statistics, respectively

**Table 2:** Table of polymorphism statistics of nuclear (nuDNA) and mitochondrial (mtDNA) DNA concatenated sequences for *M. fructicola* (MF) and *M. laxa* (ML). Number of sequences indicates the amount of sequences run in these analyses, different for each haplotype and location

	nuDNA (MF&ML)	nuDNA (MF)	nuDNA (ML)	mtDNA (MF)	mtDNA (ML)
Number of sequences	94	55	39	76	45
Polymorphic sites	144	25	5	174	7
Total number of mutations	46	25	5	174	7
Parsimony informative sites	136	18	3	174	7
Nucleotide diversity, Pi	0.04961	0.0047	0.00083	0.01962	0.00037

As a result of recombination analyses, the score of the PHI-test was 3.06E-02 for concatenated mtDNA sequences, indicating evidence of potential recombination. The only recombination event suggested was for 14_MF_Ita as recombinant and 20_ML_Ita as minor parent, with 15 M*. fructicola* major parents from different countries. Three algorithms out of six (GENECONV, Bootscan, 3Seq) supported this recombination event. Breakpoint positions were 5293 bp to 5623 bp which starts towards the end of the mobile intron region for *NAD5* and ends at the downstream region after covering 140 bp. Nuclear DNA data did not give any recombination signal.

### Phylogenetics of *M. fructicola* and *M. laxa*

**Fig. 1:**
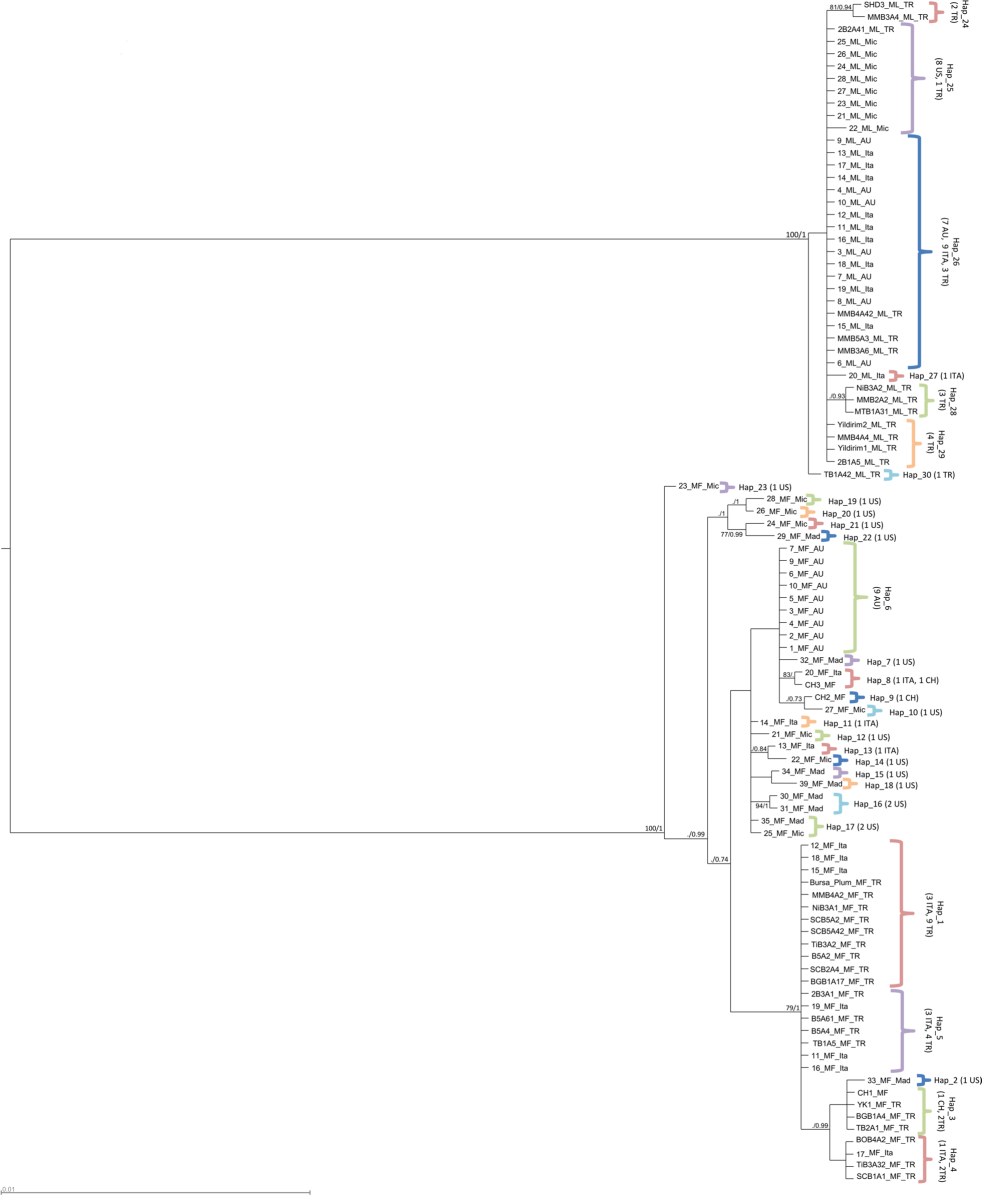
Phylogenetic tree of nuDNA concatenated sequences for *M. fructicola* and *M. laxa*. Bootstrap support values (ML) and Bayesian posterior probabilities (BI) are given at each node. ML values below 70% and BI values below 0.70 were not included. Scale bar indicates 0.01 substitutions per site. A mid-point rooting was enforced. Names of nodes contain haplotype numbers with the location they were obtained from. Numbers in brackets indicate the number of sequences per location. MF: *M. fructicola*; ML: *M. laxa*; Ita: Italy, TR: Turkey; AU: Australia; CH: China; USA: United States of America (Mad: Madeline/California/USA, Mic: Michigan/USA)

For nuDNA regions, all phylogenetic trees recovered two main clades, which corresponded to *M. fructicola* and *M. laxa*, respectively. Resolution within these two main clades varied depending on the gene, and the most complex tree structure belonged to the region *TEF1*, with several small clades both in *M. laxa* and *M. fructicola* (Additional file 1: Fig. S1, Additional file 2: Fig. S2, Additional file 3: Fig. S3). The phylogenetic tree inferred from the concatenated nuDNA dataset showed two main clades, similar to single region trees, and several subclades within each of the two species. These subclades were independent from the origin of the country (Fig. 1).

**Fig. 2:**
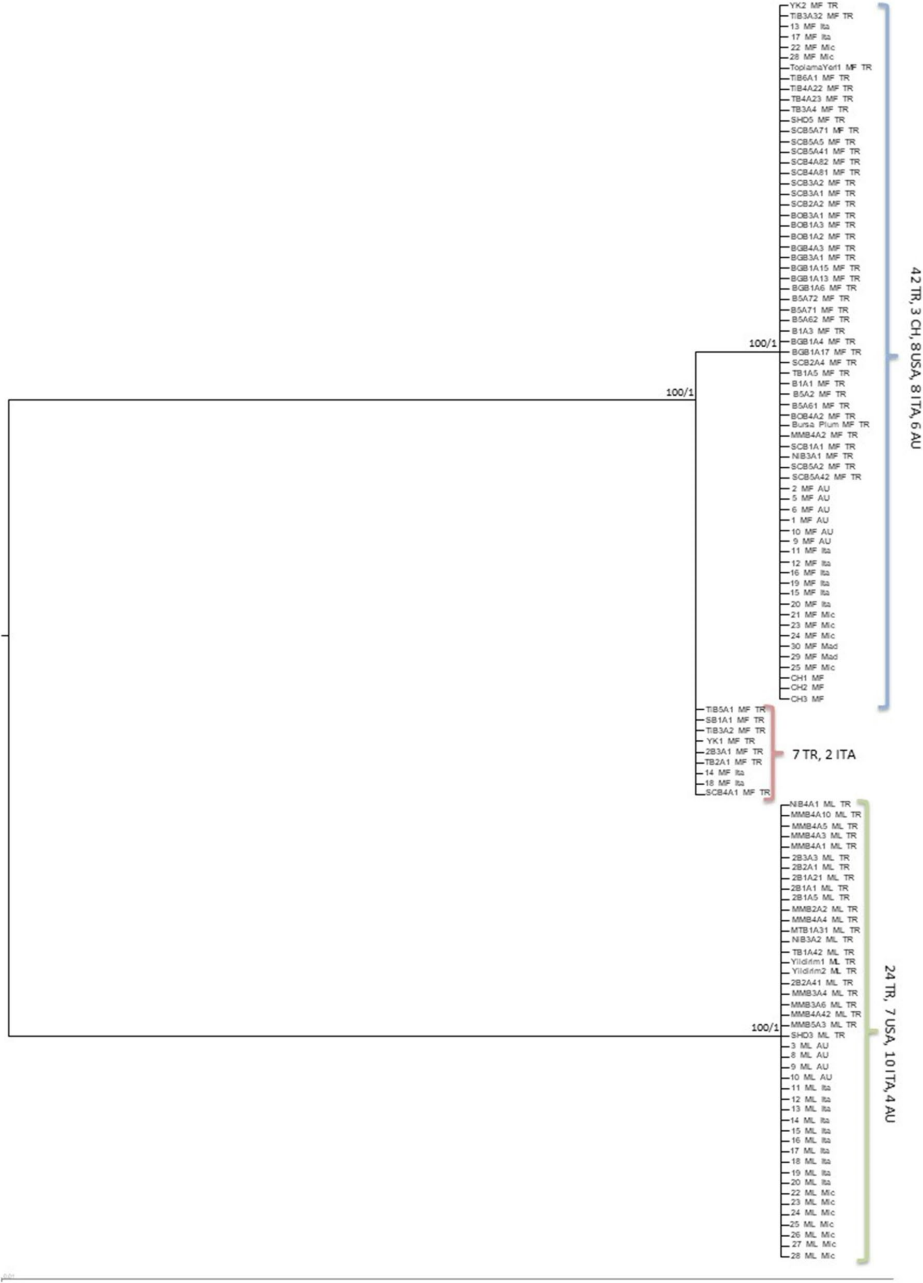
Phylogenetic tree of mtDNA concatenated sequences for *M. fructicola* and *M. laxa*. Bootstrap support values (ML) and Bayesian posterior probabilities (BI) are given at each node. ML values below 70% and BI values below 0.70 are not included. Scale bar indicates 0.01 substitutions per site. A mid-point rooting was enforced. Names of nodes contain haplotype numbers with the location they were obtained from. Numbers in brackets indicate the number of sequences per location. MF: *M. fructicola*; ML: *M. laxa*; Ita: Italy, TR: Turkey; AU: Australia; CH: China; USA: United States of America (Mad: Madeline/California/USA; Mic: Michigan/USA)

For mtDNA regions, *Cytb* only consisted of two main clades, corresponding to each species, as expected because it provided monomorphic data (Additional file 4: Fig. S4). ML and BI approaches resolved only species clades for *NAD2* (Additional file 5: Fig. S5). However, the MP tree presented a different topology for *NAD2* as those *M. fructicola* isolates carrying an intron grouped together with *M. laxa* isolates, which also had the same intron (Additional file 6: Fig. S6). All trees (MP, ML, BI) for *NAD5* grouped intron carrying isolates of *M. fructicola* together with *M. laxa* isolates, which also had the same intron (Additional file 7: Fig. S7, Additional file 8: Fig. S8). In addition, a 61 bp sequence downstream of the *NAD5* intron was identical (100%) between *M. fructicola* isolates with intron and *M. laxa*. On the other hand, this region between *M. fructicola* isolates having and not having the intron had 7 bp differences. The tree from the concatenated mitochondrial DNA data set presented two clusters within *M. fructicola* and clustered all the isolates of *M. laxa* (Fig. 2).

## Discussion

Molecular phylogenies of brown rot pathogen species *M. fructicola* and *M. laxa* were evaluated based on three nuclear and three mitochondrial gene regions in a large *Monilinia* collection collected from Turkey and including isolates from all over the world. For the first time, regions of *Calmodulin*, *SDHA*, *NAD2,* and *NAD5* genes were used as markers in a phylogenetic study for these species. Mitochondrial and nuclear DNA phylogenies of these species were also assessed comparatively.

Every region except *NAD5* (and *NAD2* with MP) in this study separated *M. fructicola* and *M. laxa* species into their own respective clades in the inferred phylogenetic trees. In the literature, differences in gene sequence data (such as *beta-tubulin*, glyceraldehyde*-triphosphate-dehydrogenase*, *RNA-binding protein* gene, *TEF1*, *hsp60*, *acp1,* and *pac1*) have been used to distinguish and name novel species within the genus *Monilinia* [11, 15, 21]. While *Monilinia* species have been successfully separated by using many protein-coding genes in phylogenetics, resolving intraspecific variation and determining lineage differences within the different species is quite challenging and requires sensitive selection of phylogenetically informative genes. Besides, as seen in many phylogeny studies including this one, the levels of polymorphism presented by each gene and the different evolutionary trajectories of the genes result in incongruence between gene trees, even within a species. Among the regions used in this study, except *Cytb*, sequences from all regions presented variation within either one or both species. Among nuDNA regions, *SDHA* and *TEF1* regions were polymorphic for both species, but *Calmodulin* regions were only polymorphic for *M. fructicola* and monomorphic for *M. laxa*. Similar to our findings, a recent study using the *TEF1* region concatenated with ITS also detected high levels of polymorphism for *M. fructicola* and revealed six phylogenetic groups [15]. While most of these groups included samples from different countries, two included only isolates from Brazil, which was represented by large number of samples in the study [15]. *NAD2* and *NAD5* regions were selected for this study due to the presence of a mobile intron, and the trees were constructed considering the presence or absence of it. All *M. laxa* isolates carried this mobile intron, and those *M. fructicola* isolates carrying the intron clustered together with the *M. laxa* clade for the *NAD2* gene in the MP analyses and for the *NAD5* gene in all phylogenetic analyses. On the other hand, *M. fructicola* isolates without intron were clustered together as a distinct clade in the *NAD2* MP tree and in all *NAD5* trees. In addition to sharing mobile introns, a sequence part at the downstream region of the mobile intron in the *NAD5* suggests that the intron may have carried a portion of the flanking sequences when moving. In addition, only *NAD5* did not show species distinction among the studied isolates and should be carefully evaluated in phylogeny studies.

When the data from mitochondrial and nuclear gene sets were compared, the nuclear gene set exhibited more variation within the species, and the nucleotide and haplotype diversity was higher. Although more mutation accumulation is expected in mitochondrial genes considering factors such as replication errors, error repair mechanism differences, and possible mobile intron mobility [33–35], mitochondrial genes are highly conserved in the studied species of *Monilinia* and it was determined that the only source of variation that could be found was an indel polymorphism related to intron insertion. In the literature, many results are indicating that the mitochondrial mutation rate is much higher than the nuclear genome mutation rate, especially in animal groups [23], however, this situation seems to be variable in fungal systems. An older study reports that mitochondrial mutation rate in fungal groups is lower than in animal groups, by comparing nuclear and mitochondrial gene polymorphisms analysed in different taxa of yeast and mammalian species [36].

It was also presented in a review that fungal mitochondrial variations are lower than variations in plant and animal groups according to the RFLP- and various multi-gene sequencing-based studies [37]. However, different pieces of evidence have recently been announced. For example, a different study on fungal species *Rhynchosporium* shows that the mitochondrial mutation rate is 77 times higher than the nuclear mutation rate [22]. They reached this conclusion as a result of ML-based phylogeny analyses and nucleotide diversity calculations using the combined sequence of thirteen mitochondrial core genes and the combined sequence of three nuclear genes (*alpha-tubulin*, *beta-tubulin,* and *ITS*) which were previously reported by Zaffarano et al. [22, 38]. While protein-encoding genes and amino-acids in the mitogenomes of *M. fructicola* and *M. laxa* were shown as highly conserved within the species, accessory regions including mobile introns were shown as the major source of the variation in the mitochondrial genomes of these species [39]. Our observations suggests that mtDNA mutation rate varies greatly in fungal species, which can create biases for marker gene selection for mtDNA to be used in phylogenetic analyses.

Recombination detection presented a single weak signal. Only recombination was proposed between an *M. laxa* isolate coded 20_ML_Ita from Italy as major parents and recombinant for another Italian isolate coded 14_MF_Ita which belongs to *M. fructicola*. However, this signal was not supported by every algorithm used for recombination detection. Suggested recombination was obtained due to the *NAD5* region, where the downstream region of the intron in *M. fructicola* aligns with *M. laxa*. Even though sexual reproduction is indirectly expected in these populations [5, 40, 41], no in vivo/vitro study have been carried out to observe inter or intraspecies sexual reproduction yet. However, hybrid species have been detected within the *Botrytis* genus [42] therefore further study is needed to cover on possible recombination status between different *Monilinia* species as well as observation of intraspecific sexual reproduction. A possible hybridization may help us understand movements of mobile elements better, as well as integrating mitochondrial mobile element detection into phylogenetic studies of fungal species.

This study included samples from five countries on four continents and different hosts. However, the host origin of the samples did not correlate with recovered clades and subclades. Clustering based on country was mainly observed in Australian samples as every sample were clustered together for each species but were not observed as strictly for other countries. Around the world, *M. fructicola* showed more diversity than *M. laxa* species, which should be taken into consideration because *M. fructicola* is a quarantine pathogen. Samples from the USA were recognized as quite variable and found in all represented subclades in the trees. In previous, studies, it has been shown that samples from the USA have high genetic diversity [43–45]. This can be an indication of the origin and diversity center of the pathogen. On the other hand, while only a small number of samples were from China, these isolates presented variations among themselves and were represented with different haplotypes based on *Calmodulin* and *TEF1* data. This diversity supports the coevolution of the host–pathogen hypothesis [46–48] as many stone and pome fruits that are the host of *Monilinia* pathogens are known to be from Asia [49–51]. While this can indicate pathogen origin as well, a larger sample set from China and/or around Asia is needed to be informative enough about diversification of these pathogens.

## Conclusions

Due to different evolutionary rates, gene regions may show various levels of nucleotide diversity, which in turn provide phylogenetic information and power of resolution at different taxonomic levels. Hence, the selection of gene regions should be carefully planned in a phylogenetic study. Although some gene regions are widely used in the literature for species identification, they may not be suitable or powerful enough to resolve phylogenies. In this study, new gene regions that can be used in phylogenetic studies of two widely known brown rot pathogen *Monilinia* species were explored, and newly designed primers were presented. Phylogenies of these two pathogens from a worldwide collection (with particular emphasis on the isolates from Turkey) were evaluated according to both nuclear and mitochondrial gene regions. We identified three nuclear gene regions with different resolution, *Calmodulin*, *SDHA* and *TEF1*, and found these regions to be more informative in phylogenetic analyses compared to mitochondrial gene regions, *NAD2*, *NAD5* and *Cytb*. Therefore, it is suggested that nuclear genes may be more informative to resolve phylogenetic branching and the discovery of new phylogenetic lineages for these species. In this regard, the numerous ongoing genomic projects on *Monilinia* will be an excellent source of new nuclear markers. On the other hand, we observed mitochondrial mobile intron effects in phylogenetic constructions. In these species, whose mitochondrial genomes are especially rich in intron and mobile intron content, how mobile introns affect the variation and phylogenetic relationships within and between pathogenic species has emerged as a very interesting research question. Besides, as mitochondrial gene mutation rates vary between fungal species, phylogenetic inference with mitochondrial genes and elements should be tested for such studies carefully. With the possible detection of a recombination event, a further study on sexual reproduction and hybridization of *Monilinia* species is needed, which can also be linked to the origin and evolution of mobile elements.

## Materials and methods

### Isolate selection and DNA extraction

*Monilinia* isolates from various locations and countries were used in this study (Additional file 9: Table S1). Turkish (TR) isolates from peach and plum fruits included 51 of *M. fructicola* and 24 of *M. laxa*, and were chosen from our large collection [41]. A total of 20 Italian (Ita) *Monilinia* isolates were kindly provided by Prof. Francesco Faretra (Dipartimento Di Scienze Del Suolo, Della Pianta E Degli Alimenti, Italy). A total of 27 isolates from the United States of America (USA) were provided by Dr. Sydney Everhart (University of Connecticut, Woodstock, Connecticut, USA) and Prof. Harald Scherm (University of Georgia, USA). Three isolates from China (CH) were provided by Prof. Chaoxi Luo (Huazhong Agricultural University, China) and 20 isolates from Australia (AU) were provided by Dr. Stephen J. Wylie (Western Australian State Agricultural Biotechnology Centre, Australia). Information about the names, hosts, and locations of all isolates is provided in Additional file 9: Table S1.

Foreign samples were provided either as cultures on filter papers or DNA samples. Turkish isolates from the storage at − 20 °C and all the foreign isolates sent as a culture were revitalized at 23 °C on Potato Dextrose Agar (PDA) media. Species identification of all Turkish isolates was through morphological assessments and PCR reactions with species-specific markers [41]. Then, the isolates were transferred to potato dextrose broth (PDB) media and incubated for 7 days in a rotary shaker at room temperature. Mycelia were harvested and collected through vacuum filtration. DNA extraction was performed with the Fungi/Yeast Genomic DNA Isolation Kit (Norgen, Canada). Concentrations of extracted DNA samples, as well as of foreign samples directly sent as DNA, were determined using the Implen Nanophotometer P330.

### Selecting nuclear and mitochondrial gene regions for phylogenetic analyses

In preliminary analyses, many nuclear gene regions (including *beta-tubulin, glyceraldehyde 3-phosphate dehydrogenase, internal transcribed spacer, translation elongation factor 1 alpha, calmodulin, heat shock protein 60, polygalacturonase 1* and *2, necrosis* and *ethylene-inducing protein precursor 1 and precursor 2, Calmodulin, succinate dehydrogenase* subunits*, RNA polymerase II subunit, multidrug resistance regulator 1, ATPase* and mating type genes) were checked for phylogenetic information within and between *Monilinia* species. Sequence data of all mentioned regions were retrieved from NCBI and mapped on full genome data of 8 *M. fructicola* and 8 *M. laxa* isolates [39], which are available under Bioproject PRJNA846280. Among nuclear regions, the *translation elongation factor 1 alpha* (*TEF1*) was selected as it is a commonly used region in fungal phylogenetic studies including *Monilinia* pathogens and many other fungal groups [11, 52, 53]. *Calmodulin* has been also shown as phylogenetically informative for different fungal species [54, 55], however, this study was the first to use the *Calmodulin* region for phylogenetic inferences in *Monilinia* species. While the *SDHA* region has been isolated before and characterized with different mutations in a few isolates of *M. fructicola* [56], it will also be the first time for it to be included in a phylogenetic study. All four SDH genes were evaluated, and the partial sequence of the *SDHA* gene was chosen as the one that maximizes phylogenetic information.

Complete mitochondrial genomes of both species were characterized previously for 16 isolates [39, 41]. Among mitochondrial regions, *cytochrome b* is a well-known gene that has been used in phylogenetic analysis [11]. Moreover, *NAD2* and *NAD5* regions were characterized very recently from *M. laxa* and *M. fructicola* [32, 39], and were chosen for the present study because they show intron size differences within and in between *Monilinia* species.

### Primer design

**Fig. 3:**
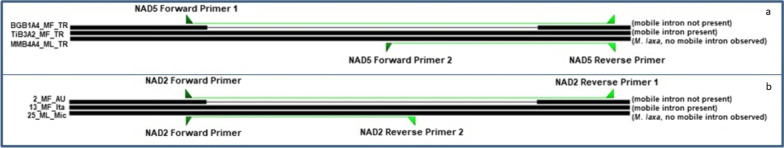
**a** Two *M. fructicola* and a *M. laxa* sequences of *NAD5* region, while mobile intron in not present in the first sequence (BGB1A4_MF_TR), the second sequence (TiB3A2_MF_TR) has the mobile intron, whereas mobile intron is not observed for *M. laxa* sequences. While *NAD5* Reverse Primer binds to the same site, *NAD5* Forward Primer 2 only binds to mobile intron sequence. **b** NAD2 forward primer also binds to upstream region of possible mobile intron presence, while Reverse Primer 1 binds to downstream region of this site, Reverse Primer 2 binds into mobile intron if present. Similarly, *M. laxa* does not show and mobile intron presence for NAD2

Nearly universal TEF primers for fungi designed by Carbone and Kohn [52] worked properly for for *M. laxa* but not for all *M. fructicola* isolates due to few mutations in the primer binding sites. Hence, new PCR primers were generated to amplify each gene region in this study except *TEF1* in *M. laxa.* Primers for nuclear genes were designed from whole genome sequence data for both species and those for mitochondrial genes were obtained from whole mitochondrial genomes [32, 39]. Primers were designed using Primer3 [57] and Geneious 9.1.8 [58]. Primer information is presented in Additional file 10: Table S2. Since *NAD5* and *NAD2* regions were chosen due to their intron size differences among isolates of *M. fructicola*, three primers were designed for each gene considering presence or absence of the intron (Additional file 10: Table S2 and Fig. 3). Primers designed for *M. fructicola* sequences having mobile intron insertion also worked on *M. laxa* isolates as they all had intron presence.

### PCR and sequencing

For both species, *SDHA*, *Calmodulin*, *NAD2*, *NAD5,* and *TEF1* regions were amplified with the following protocol: 7 min at 94 °C, 35 cycles of 45 s at 94 °C, 45 s at 57 °C, 45 s at 72 °C and, 10 min of 72 °C of final extension. For *Cytb*, the same protocol was applied but using 59 °C as the annealing temperature. For all PCR reactions, the same 20 µL reaction mixture was used: 1Χ PCR Buffer without MgCl_2_ (Fermentas, Thermo Fisher Scientific), 2 mM MgCl_2_, 0.2 mM dNTPs, 0.4 µM of each primer, 1U of DreamTaq DNA Polymerase (Thermo Fisher Scientific), and 0.1 µL of DNA template. PCR products were run on 1.5% agarose gel, stained with 5 µL/100 mL of SafeView (Applied Biological Materials Inc., Canada) dye, and viewed on a UV transilluminator (UVP Model M-10E Mini, Thermo Science). Successfully amplified products were sent for Sanger sequencing with an ABI 3500xL Genetic Analyzer (Applied Biosystems, MedSanTek Lab, Turkey). While intron size differences were perceptible on the gel for *NAD2* and *NAD5*, some samples were sequenced as well. Raw sequence data were checked and trimmed using Geneious v.9.1.8. [58]. Each polymorphism was checked manually by re-checking nucleotide peaks. Multiple sequence alignments for each gene were performed using the ClustalW algorithm. The concatenated data sets for nuclear and mitochondrial genes were generated with Geneious v.9.1.8. [58]. Fasta files were obtained for each data set and converted into Nexus files using Mesquite v3.61 [59] for further analysis.

### Phylogenetic analyses

Phylogenetic trees were reconstructed using maximum parsimony (MP), maximum likelihood (ML), and Bayesian inference (BI). Best-fit evolutionary models for each gene as well as for each concatenated data set were calculated using MrModelTest 2.2 [60] and jmodeltest2 [61], and are provided in Additional file 11: Table S3. BI analyses were run using MrBayes v3.2.7 [62]. For every analysis, 1,000,000 generations were run with 4 Markov chains, and trees were sampled every 1000 generations. The first 25% of trees sampled were discarded as burn-in. ML analyses were run on RAxmlGUI 2.0 [63]. For datasets with GTR substitution rate, raxmlHPC binary was used. ML + rapid bootstrap analyses were done with 1000 replications with randomized seeds. For datasets analysed under other evolutionary models, the raxml-ng binary was used. ML + thorough bootstrap + consensus analyses were run with 1000 replications with randomized seeds. PAUP* [64] was used for MP analyses. For each analysis, 1000 bootstrap replications were done with randomized seeds. Gaps were set as 5th state to interpret introns indels in *NAD2* and *NAD5* regions. No outgroup was used due to highly divergent sequences in available closely related fungal taxa. Instead, inferred trees were mid-point rooted.

Only haplotypes from each country were represented on the trees with the number of isolates represented by each haplotype in parentheses. Although the same haplotypes have been detected in different countries, the haplotypes are shown by country basis. Trees were visualized using FigTree v1.4.4 [65] and Archaeopteryx [66]. Both ML and BI trees obtained in this study recovered the same topologies, therefore, BI phylograms were used for visualisation. On all trees, only ML bootstrapped values higher than 70% and BI probability values higher than 0.7 were shown. MP analysis based on *NAD2* and all trees based on *NAD5* arrived at a different tree topology due to the presence of an intron in some *M. fructicola* and all *M. laxa* isolates, and thus, they were represented separately.

### Nucleotide diversity and recombination test

DNA polymorphism statistics for each region and concatenated data sets were obtained using DnaSP v6.12.03 [67]. Recombination tests were performed with the Recombination Detection Program (RDP4) [68], using the concatenated nuDNA and mtDNA datasets as input. Algorithms GENECONV, Bootscan, 3Seq, RDP, Maxchi, and Chimaera were applied in the recombination analyses.

## Supplementary Information

**Additional file 1: Figure S1.** Phylogenetic tree of *Calmodulin* regions for *M. fructicola* and *M. laxa* sequences. Analyses were done with Maximum Likelihood analyses on RAxML and Bayesian Inference with MrBayes. Bootstrap support values (ML) and Bayesian posterior probabilities (BI) are given at each node. Scale bar indicates number of substitutions per site. Phylogram is mid-pointed. Names of nodes contain haplotype numbers with the location they were obtained from. Numbers in brackets indicate the number of sequences that node originally has.

**Additional file 2: Figure S2.** Phylogenetic tree of *SDHA* regions for *M. fructicola* and *M. laxa* sequences. Analyses were done with Maximum Likelihood analyses on RAxML and Bayesian Inference with MrBayes. Bootstrap support values (ML) and Bayesian posterior probabilities (BI) are given at each node. Scale bar indicates number of substitutions per site. Phylogram is mid-pointed. Names of nodes contain haplotype numbers with the location they were obtained from. Numbers in brackets indicate the number of sequences that node originally has.

**Additional file 3: Figure S3.** Phylogenetic tree of *TEF1* regions for *M. fructicola* and *M. laxa* sequences. Analyses were done with Maximum Likelihood analyses on RAxML and Bayesian Inference with MrBayes. Bootstrap support values (ML) and Bayesian posterior probabilities (BI) are given at each node. Scale bar indicates number of substitutions per site. Phylogram is mid-pointed. Names of nodes contain haplotype numbers with the location they were obtained from. Numbers in brackets indicate the number of sequences that node originally has.

**Additional file 4: Figure S4.** Phylogenetic tree of *Cytb* regions for *M. fructicola* and *M. laxa* sequences. Analyses were done with Maximum Likelihood analyses on RAxML and Bayesian Inference with MrBayes. Bootstrap support values (ML) and Bayesian posterior probabilities (BI) are given at each node. Scale bar indicates number of substitutions per site. Phylogram is mid-pointed. Names of nodes contain haplotype numbers with the location they were obtained from. Numbers in brackets indicate the number of sequences that node originally has.

**Additional file 5: Figure S5.** Phylogenetic tree of *NAD2* regions for *M. fructicola* and *M. laxa* sequences. Analyses were done with Maximum Likelihood analyses on RAxML and Bayesian Inference with MrBayes. Bootstrap support values (ML) and Bayesian posterior probabilities (BI) are given at each node. Scale bar indicates number of substitutions per site. Phylogram is mid-pointed. Names of nodes contain haplotype numbers with the location they were obtained from. Numbers in brackets indicate the number of sequences that node originally has.

**Additional file 6: Figure S6.** Phylogenetic tree of *NAD2* regions for *M. fructicola* and *M. laxa* sequences. Analyses were done with Maximum Parsimony on PAUP. Tree is mid-point rooted. Names of nodes contain haplotype numbers with the location they were obtained from. Numbers in brackets indicate the number of sequences that node originally has.

**Additional file 7: Figure S7.** Phylogenetic tree of *NAD5* regions for *M. fructicola* and *M. laxa* sequences. Analyses were done with Maximum Likelihood analyses on RAxML and Bayesian Inference with MrBayes. Bootstrap support values (ML) and Bayesian posterior probabilities (BI) are given at each node. Scale bar indicates number of substitutions per site. Phylogram is mid-pointed. Names of nodes contain haplotype numbers with the location they were obtained from. Numbers in brackets indicate the number of sequences that node originally has.

**Additional file 8: Figure S8.** Phylogenetic tree of *NAD5* regions for *M. fructicola* and *M. laxa* sequences. Analyses were done with Maximum Parsimony on PAUP. Tree is mid-point rooted. Names of nodes contain haplotype numbers with the location they were obtained from. Numbers in brackets indicate the number of sequences that node originally has.

**Additional file 9: Table S1**. Table of isolate Codes, Names, Species, Hosts, Location, Mating Type and Haplotypes of each gene for all isolates used in this study.

**Additional file 10: Table S2.** Table of every primer used in this study.

**Additional file 11: Table S3.** Table of each regions substitution rates. Outer left column shows used software, second column indicates algorithms used.

## Data Availability

Sequences representing each haplotype of the studied nuclear gene region were deposited to NCBI GenBank as: OP036604-OP036610 for Calmodulin; OP090593-OP090612 for SDHA; OP090613-OP090640 for TEF1. Any further data that support the findings of this study are available from the corresponding author upon reasonable request
