# Supplementary figures and images for: Phylogenetic divergences in brown rot fungal pathogens of Monilinia species from a worldwide collection: inferences based on the nuclear versus mitochondrial genes
Source: BMC Ecol Evol. 2022 Oct 21;22:119. doi: 10.1186/s12862-022-02079-6 (PMC9585774; doi:10.1186/s12862-022-02079-6)

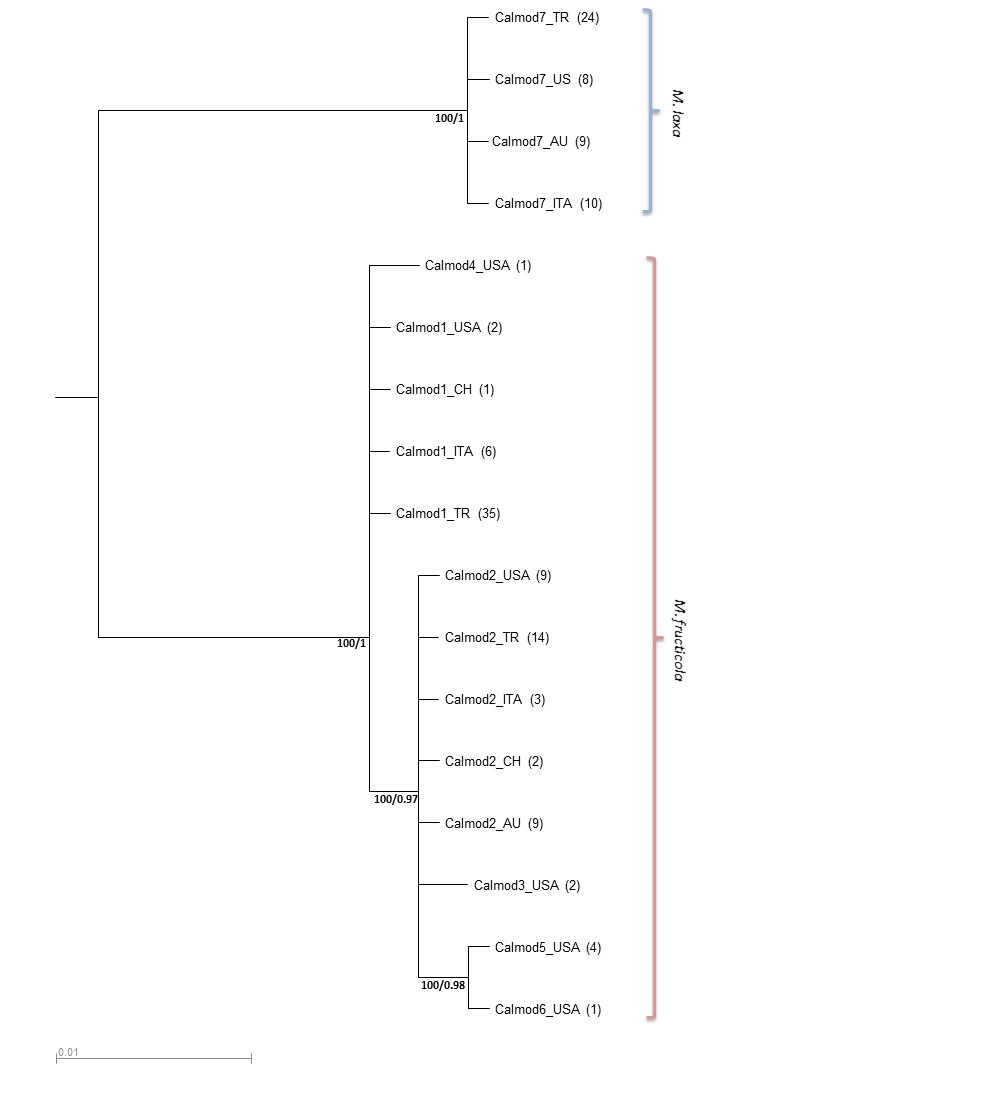

Supplement: Supplementary file 1 — Additional file 1: Figure S1. Phylogenetic tree of Calmodulin regions for M. fructicola and M. laxa sequences. Analyses were done with Maximum Likelihood analyses on RAxML and Bayesian Inference with MrBayes. Bootstrap support values (ML) and Bayesian posterior probabilities (BI) are given at each node. Scale bar indicates number of substitutions per site. Phylogram is mid-pointed. Names of nodes contain haplotype numbers with the location they were obtained from. Numbers in brackets indicate the number of sequences that node originally has. [file 12862_2022_2079_MOESM1_ESM.tiff]

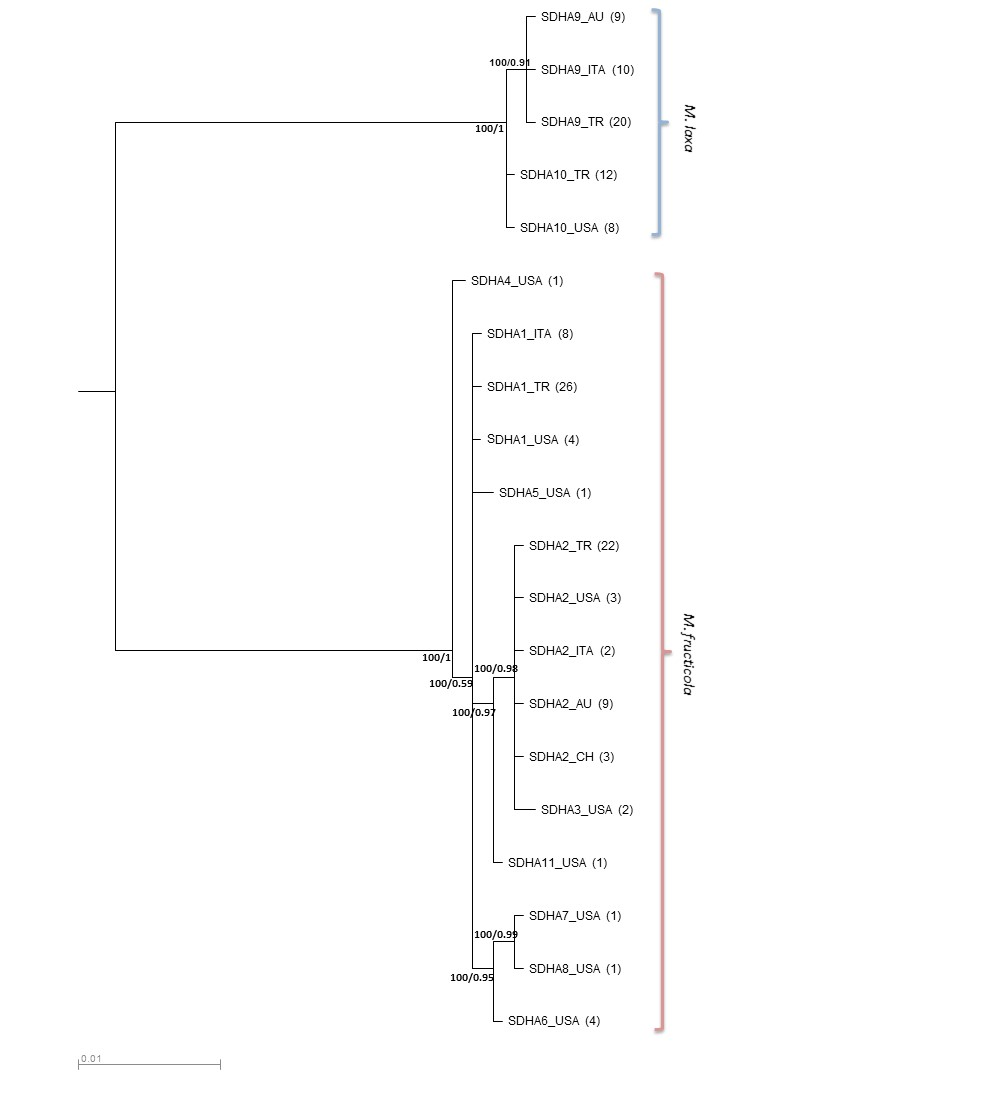

Supplement: Supplementary file 2 — Additional file 2: Figure S2. Phylogenetic tree of SDHA regions for M. fructicola and M. laxa sequences. Analyses were done with Maximum Likelihood analyses on RAxML and Bayesian Inference with MrBayes. Bootstrap support values (ML) and Bayesian posterior probabilities (BI) are given at each node. Scale bar indicates number of substitutions per site. Phylogram is mid-pointed. Names of nodes contain haplotype numbers with the location they were obtained from. Numbers in brackets indicate the number of sequences that node originally has. [file 12862_2022_2079_MOESM2_ESM.tiff]

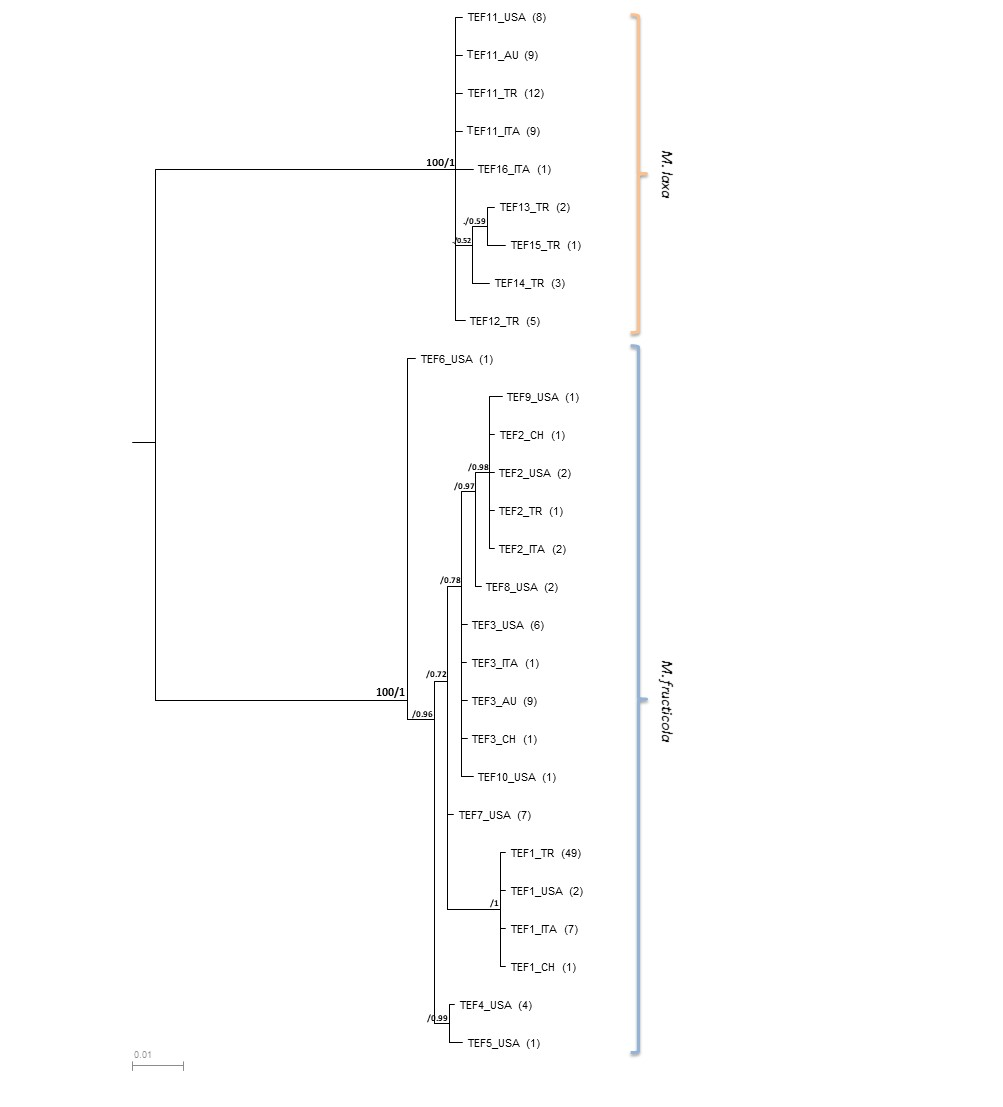

Supplement: Supplementary file 3 — Additional file 3: Figure S3. Phylogenetic tree of TEF1 regions for M. fructicola and M. laxa sequences. Analyses were done with Maximum Likelihood analyses on RAxML and Bayesian Inference with MrBayes. Bootstrap support values (ML) and Bayesian posterior probabilities (BI) are given at each node. Scale bar indicates number of substitutions per site. Phylogram is mid-pointed. Names of nodes contain haplotype numbers with the location they were obtained from. Numbers in brackets indicate the number of sequences that node originally has. [file 12862_2022_2079_MOESM3_ESM.tiff]

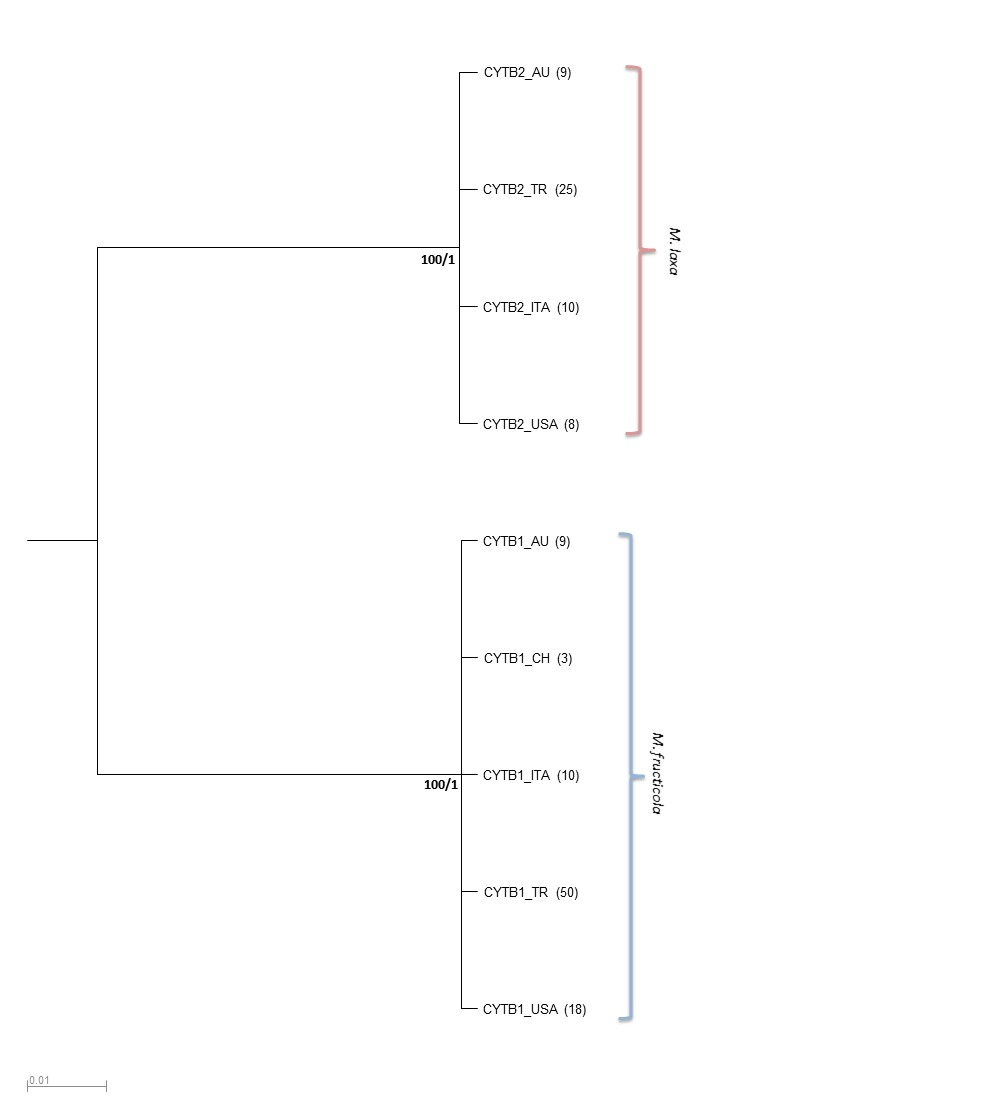

Supplement: Supplementary file 4 — Additional file 4: Figure S4. Phylogenetic tree of Cytb regions for M. fructicola and M. laxa sequences. Analyses were done with Maximum Likelihood analyses on RAxML and Bayesian Inference with MrBayes. Bootstrap support values (ML) and Bayesian posterior probabilities (BI) are given at each node. Scale bar indicates number of substitutions per site. Phylogram is mid-pointed. Names of nodes contain haplotype numbers with the location they were obtained from. Numbers in brackets indicate the number of sequences that node originally has. [file 12862_2022_2079_MOESM4_ESM.tiff]

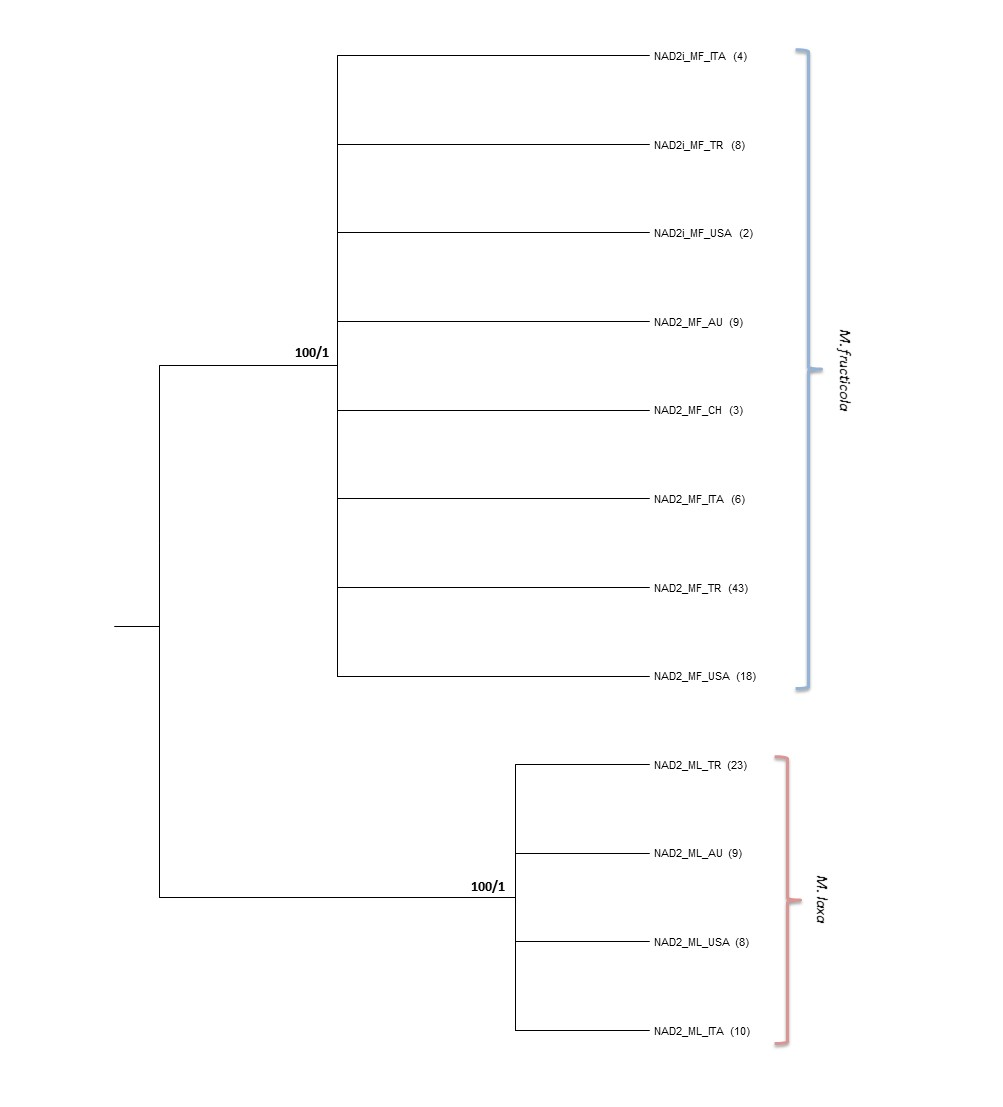

Supplement: Supplementary file 5 — Additional file 5: Figure S5. Phylogenetic tree of NAD2 regions for M. fructicola and M. laxa sequences. Analyses were done with Maximum Likelihood analyses on RAxML and Bayesian Inference with MrBayes. Bootstrap support values (ML) and Bayesian posterior probabilities (BI) are given at each node. Scale bar indicates number of substitutions per site. Phylogram is mid-pointed. Names of nodes contain haplotype numbers with the location they were obtained from. Numbers in brackets indicate the number of sequences that node originally has. [file 12862_2022_2079_MOESM5_ESM.tiff]

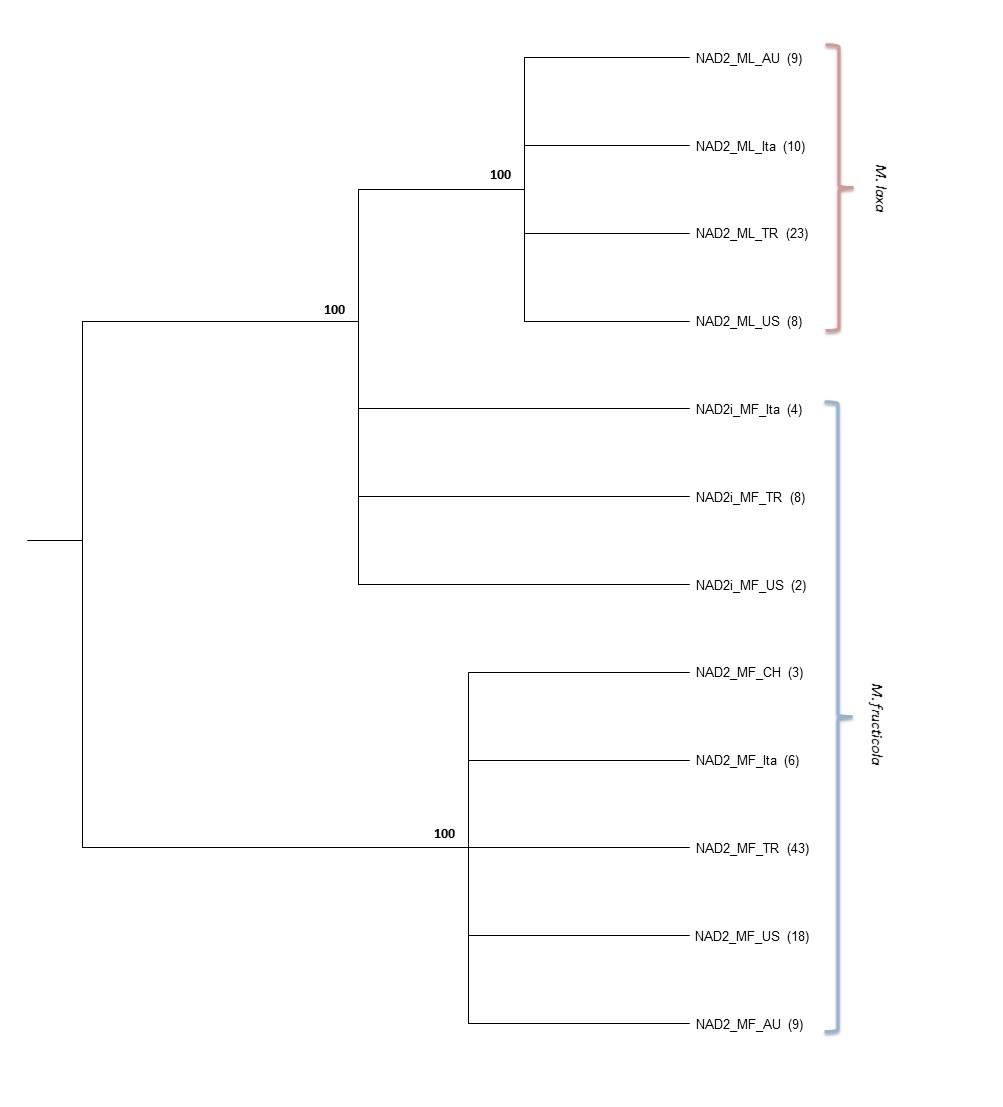

Supplement: Supplementary file 6 — Additional file 6: Figure S6. Phylogenetic tree of NAD2 regions for M. fructicola and M. laxa sequences. Analyses were done with Maximum Parsimony on PAUP. Tree is mid-point rooted. Names of nodes contain haplotype numbers with the location they were obtained from. Numbers in brackets indicate the number of sequences that node originally has. [file 12862_2022_2079_MOESM6_ESM.tiff]

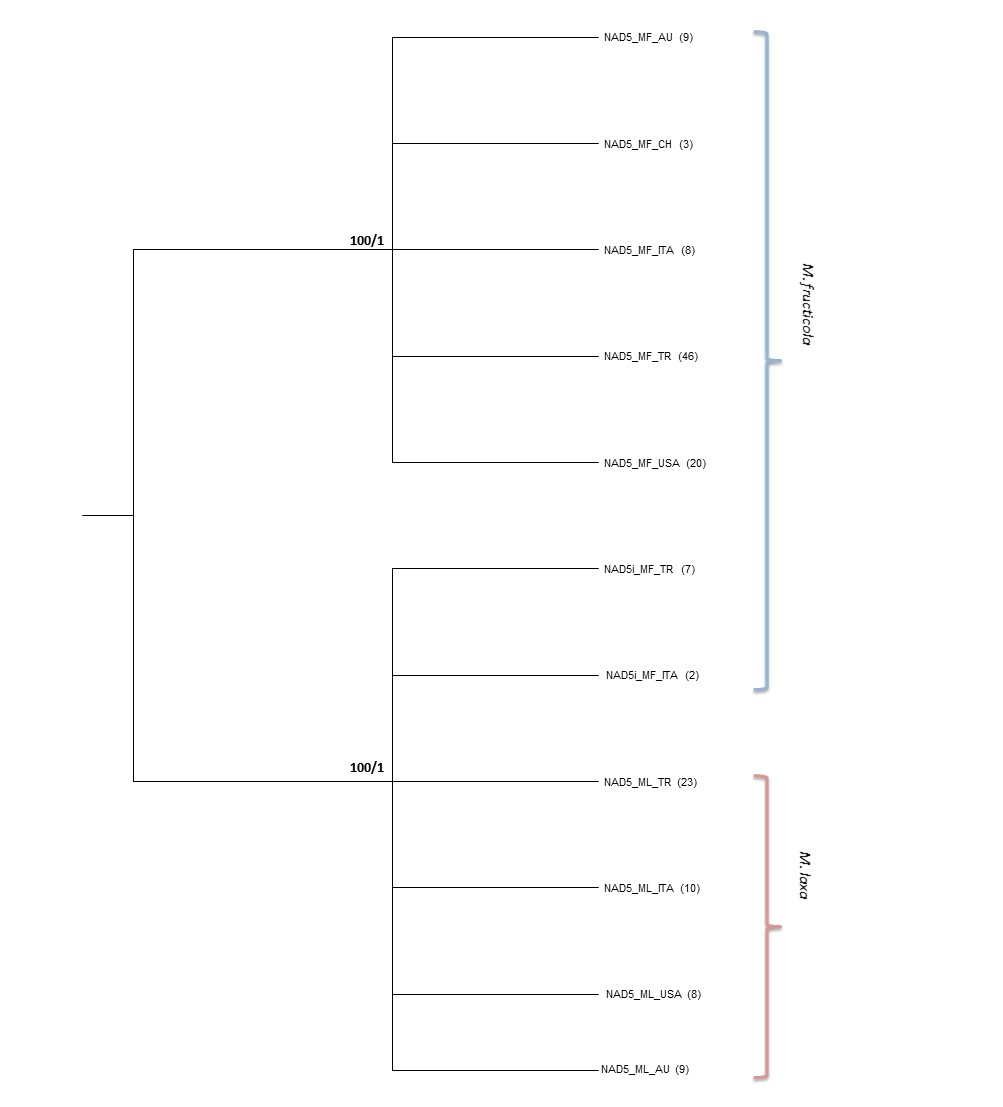

Supplement: Supplementary file 7 — Additional file 7: Figure S7. Phylogenetic tree of NAD5 regions for M. fructicola and M. laxa sequences. Analyses were done with Maximum Likelihood analyses on RAxML and Bayesian Inference with MrBayes. Bootstrap support values (ML) and Bayesian posterior probabilities (BI) are given at each node. Scale bar indicates number of substitutions per site. Phylogram is mid-pointed. Names of nodes contain haplotype numbers with the location they were obtained from. Numbers in brackets indicate the number of sequences that node originally has. [file 12862_2022_2079_MOESM7_ESM.tiff]

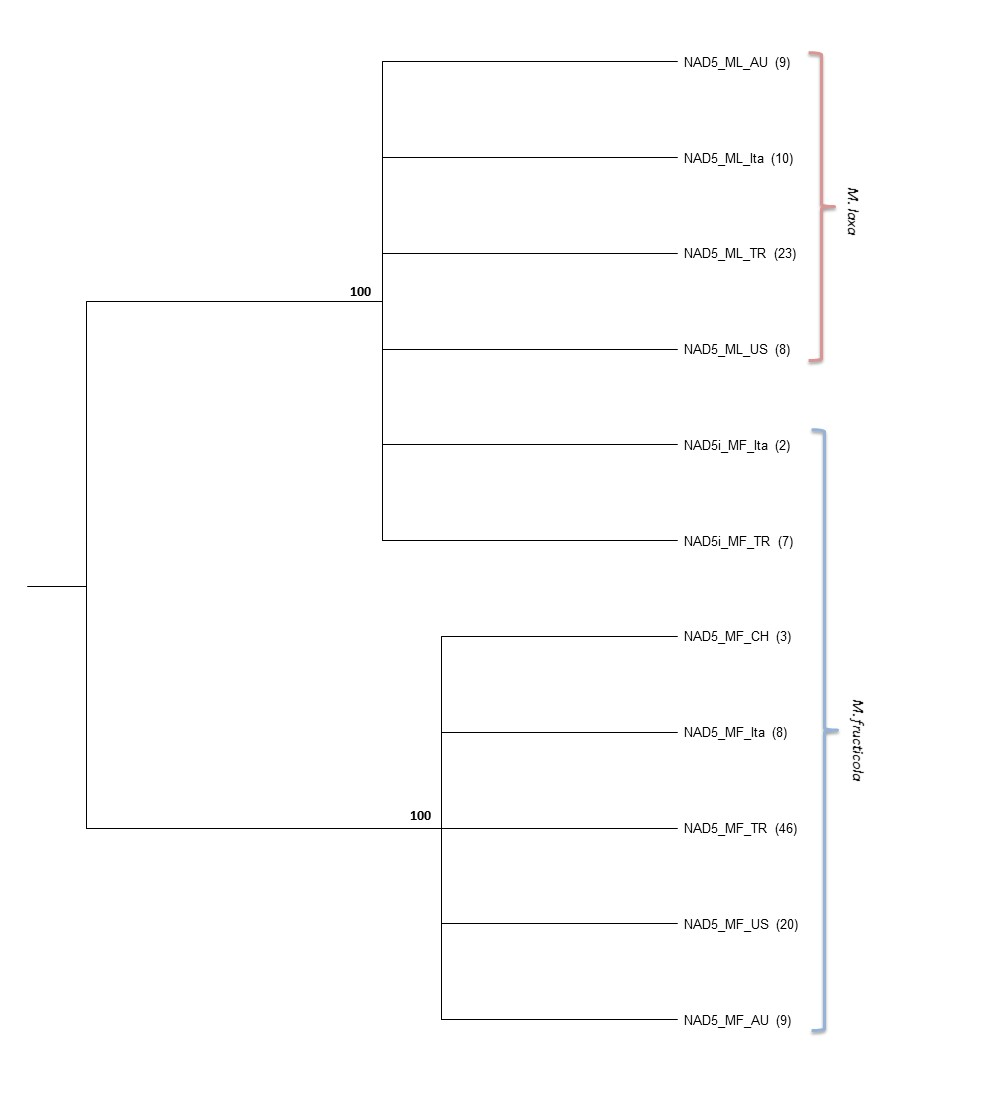

Supplement: Supplementary file 8 — Additional file 8: Figure S8. Phylogenetic tree of NAD5 regions for M. fructicola and M. laxa sequences. Analyses were done with Maximum Parsimony on PAUP. Tree is mid-point rooted. Names of nodes contain haplotype numbers with the location they were obtained from. Numbers in brackets indicate the number of sequences that node originally has. [file 12862_2022_2079_MOESM8_ESM.tiff]
