# Supplementary material for: Phylogenetic divergences in brown rot fungal pathogens of Monilinia species from a worldwide collection: inferences based on the nuclear versus mitochondrial genes
Source: BMC Ecol Evol. 2022 Oct 21;22:119. doi: 10.1186/s12862-022-02079-6 (PMC9585774; doi:10.1186/s12862-022-02079-6)
Supplement: Supplementary file 9 — Additional file 9: Table S1. Table of isolate Codes, Names, Species, Hosts, Location, Mating Type and Haplotypes of each gene for all isolates used in this study. [file 12862_2022_2079_MOESM9_ESM.docx]

Supplementary Table 1: Table of isolate Codes, Names, Species, Hosts, Location, Mating Type and Haplotypes of each gene for all isolates used in this study

| Code | Name | Species | Host | Location Info | Mating Type | Calmodulin  Haplotype | SDHA Haplotype | TEF1 Haplotype | Cytb  Haplotype | NAD2 Haplotype | NAD5 Haplotype |
| --- | --- | --- | --- | --- | --- | --- | --- | --- | --- | --- | --- |
| 2L | M25 | *M. laxa* | Unknown | Australia (AU) | MAT1-2 | Calmod7 | SDHA9 | TEF11 | Cyt_ML | NAD2L | NAD5L |
| 3L | M27 | *M. laxa* | Unknown | Australia (AU) | MAT1-1 | Calmod7 | SDHA9 | TEF11 | Cyt_ML | NAD2L | NAD5L |
| 4L | M31 | *M. laxa* | Unknown | Australia (AU) | MAT1-1 | Calmod7 | SDHA9 | TEF11 | Cyt_ML | NAD2L | NAD5L |
| 5L | M40 | *M. laxa* | Unknown | Australia (AU) | MAT1-2 | Calmod7 | SDHA9 | TEF11 | Cyt_ML | NAD2L | NAD5L |
| 6L | M50 | *M. laxa* | Unknown | Australia (AU) | MAT1-1 | Calmod7 | SDHA9 | TEF11 | Cyt_ML | NAD2L | NAD5L |
| 7L | M128 | *M. laxa* | Unknown | Australia (AU) | MAT1-1 | Calmod7 | SDHA9 | TEF11 | Cyt_ML | NAD2L | NAD5L |
| 8L | M138 | *M. laxa* | Unknown | Australia (AU) | MAT1-1 | Calmod7 | SDHA9 | TEF11 | Cyt_ML | NAD2L | NAD5L |
| 9L | M140 | *M. laxa* | Unknown | Australia (AU) | MAT1-1 | Calmod7 | SDHA9 | TEF11 | Cyt_ML | NAD2L | NAD5L |
| 10L | M141 | *M. laxa* | Unknown | Australia (AU) | MAT1-1 | Calmod7 | SDHA9 | TEF11 | Cyt_ML | NAD2L | NAD5L |
| 1F | M88 | *M. fructicola* | Unknown | Australia (AU) | MAT1-2 | Calmod2 | SDHA2 | TEF3 | Cyt_MF | NAD2 | NAD5 |
| 2F | M94 | *M. fructicola* | Unknown | Australia (AU) | MAT1-1 | Calmod2 | SDHA2 | TEF3 | Cyt_MF | NAD2 | NAD5 |
| 3F | M101 | *M. fructicola* | Unknown | Australia (AU) | MAT1-2 | Calmod2 | SDHA2 | TEF3 | Cyt_MF | NAD2 | NAD5 |
| 4F | M105 | *M. fructicola* | Unknown | Australia (AU) | MAT1-2 | Calmod2 | SDHA2 | TEF3 | Cyt_MF | NAD2 | NAD5 |
| 5F | M106 | *M. fructicola* | Unknown | Australia (AU) | MAT1-2 | Calmod2 | SDHA2 | TEF3 | Cyt_MF | NAD2 | NAD5 |
| 6F | M143 | *M. fructicola* | Unknown | Australia (AU) | MAT1-1 | Calmod2 | SDHA2 | TEF3 | Cyt_MF | NAD2 | NAD5 |
| 7F | M144 | *M. fructicola* | Unknown | Australia (AU) | MAT1-2 | Calmod2 | SDHA2 | TEF3 | Cyt_MF | NAD2 | NAD5 |
| 9F | M170 | *M. fructicola* | Unknown | Australia (AU) | MAT1-1 | Calmod2 | SDHA2 | TEF3 | Cyt_MF | NAD2 | NAD5 |
| 10F | M175 | *M. fructicola* | Unknown | Australia (AU) | MAT1-1 | Calmod2 | SDHA2 | TEF3 | Cyt_MF | NAD2 | NAD5 |
| 11F | MFRC_69 | *M. fructicola* | Cherry | Italia (ITA), Caserta | MAT1-1 | Calmod2 | SDHA1 | TEF1 | Cyt_MF | NAD2 | NAD5 |
| 12F | MFRC_93 | *M. fructicola* | Cherry | Italia (ITA), Caserta | MAT1-1 | Calmod1 | SDHA1 | TEF1 | Cyt_MF | NAD2 | NAD5 |
| 13F | MFRC_106 | *M. fructicola* | Cherry | Italia (ITA), Bisceglie (BAT) | MAT1-1 | Calmod1 | SDHA1 | TEF2 | Cyt_MF | NAD2i | NAD5 |
| 14F | MFRC_148 | *M. fructicola* | Plum | Italia (ITA), Tursi (MT) | MAT1-2 | Calmod2 | SDHA1 | TEF2 | Cyt_MF | NAD2i | NAD5i |
| 15F | MFRC_201 | *M. fructicola* | Plum | Italia (ITA), Tursi (MT) | MAT1-2 | Calmod1 | SDHA1 | TEF1 | Cyt_MF | NAD2 | NAD5 |
| 16F | MFRC_230 | *M. fructicola* | Peach plate | Italia (ITA), Tursi (MT) | MAT1-2 | Calmod2 | SDHA1 | TEF1 | Cyt_MF | NAD2 | NAD5 |
| 17F | MFRC_234 | *M. fructicola* | Peach plate | Italia (ITA), Tursi (MT) | MAT1-1 | Calmod1 | SDHA2 | TEF1 | Cyt_MF | NAD2i | NAD5 |
| 18F | MFRC_261 | *M. fructicola* | Peach plate | Italia (ITA), Policoro (MT) | MAT1-1 | Calmod1 | SDHA1 | TEF1 | Cyt_MF | NAD2i | NAD5i |
| 19F | MFRC_373 | *M. fructicola* | Peach | Italia (ITA), Loconia | MAT1-2 | Calmod2 | SDHA1 | TEF1 | Cyt_MF | NAD2 | NAD5 |
| 20F | MFRC_381 | *M. fructicola* | Peach | Italia (ITA) | MAT1-2 | Calmod1 | SDHA2 | TEF3 | Cyt_MF | NAD2 | NAD5 |
| 11L | MLAX_23 | *M. laxa* | Unknown | Italia (ITA) | MAT1-1 | Calmod7 | SDHA9 | TEF11 | Cyt_ML | NAD2L | NAD5L |
| 12L | MLAX_33 | *M. laxa* | Almonds | Italia (ITA) | MAT1-2 | Calmod7 | SDHA9 | TEF11 | Cyt_ML | NAD2L | NAD5L |
| 13L | MLAX_36 | *M. laxa* | Almonds | Italia (ITA) | MAT1-1 | Calmod7 | SDHA9 | TEF11 | Cyt_ML | NAD2L | NAD5L |
| 14L | MLAX_57 | *M. laxa* | Cherry | Italia (ITA), Conversano (BA) | MAT1-1 | Calmod7 | SDHA9 | TEF11 | Cyt_ML | NAD2L | NAD5L |
| 15L | MLAX_61 | *M. laxa* | Apricots | Italia (ITA), Casalnuovo Monterotaro (FG) | MAT1-1 | Calmod7 | SDHA9 | TEF11 | Cyt_ML | NAD2L | NAD5L |
| 16L | MLAX_63 | *M. laxa* | Apricots | Italia (ITA), Mola di Bari (BA) | MAT1-1 | Calmod7 | SDHA9 | TEF11 | Cyt_ML | NAD2L | NAD5L |
| 17L | MLAX_66 | *M. laxa* | Cherry | Italia (ITA), Acquaviva delle fonti (BA) | MAT1-1 | Calmod7 | SDHA9 | TEF11 | Cyt_ML | NAD2L | NAD5L |
| 18L | MLAX_316 | *M. laxa* | Cherry | Italia (ITA), Gioia del colle (BA) | MAT1-1 | Calmod7 | SDHA9 | TEF11 | Cyt_ML | NAD2L | NAD5L |
| 19L | MLAX_371 | *M. laxa* | Apricots | Italia (ITA), Sammichele di Bari | MAT1-1 | Calmod7 | SDHA9 | TEF11 | Cyt_ML | NAD2L | NAD5L |
| 20L | MLAX_509 | *M. laxa* | Peach | Italia (ITA) | MAT1-1 | Calmod7 | SDHA9 | TEF16 | Cyt_ML | NAD2L | NAD5L |
| 21F | 19HKB-1 | *M. fructicola* | Unknown | Michigan (US) | MAT1-2 | Calmod5 | SDHA6 | TEF2 | Cyt_MF | NAD2 | NAD5 |
| 22F | 09BR-32 | *M. fructicola* | Unknown | Michigan (US) | MAT1-1 | Calmod1 | SDHA1 | TEF9 | Cyt_MF | NAD2i | NAD5 |
| 23F | 10DRAM-2 | *M. fructicola* | Unknown | Michigan (US) | MAT1-1 | Calmod2 | SDHA4 | TEF6 | Cyt_MF | NAD2 | NAD5 |
| 24F | 11MRAM-10 | *M. fructicola* | Unknown | Michigan (US) | MAT1-1 | Calmod3 | SDHA11 | TEF4 | Cyt_MF | NAD2 | NAD5 |
| 25F | 19CDP-7 | *M. fructicola* | Unknown | Michigan (US) | MAT1-1 | Calmod2 | SDHA6 | TEF7 | Cyt_MF | NAD2 | NAD5 |
| 26F | 19DTA-12 | *M. fructicola* | Unknown | Michigan (US) | MAT1-1 | Calmod5 | SDHA1 | TEF4 | Cyt_MF | NAD2 | NAD5 |
| 27F | 19SAR3-1 | *M. fructicola* | Unknown | Michigan (US) | MAT1-2 | Calmod5 | SDHA2 | TEF2 | Cyt_MF | NAD2 | NAD5 |
| 28F | 19DRK-15 | *M. fructicola* | Unknown | Michigan (US) | MAT1-2 | Calmod6 | SDHA6 | TEF4 | Cyt_MF | NAD2i | NAD5 |
| 21L | 130MP-6B | *M. laxa* | Unknown | Michigan (US) | MAT1-1 | Calmod7 | SDHA10 | TEF11 | Cyt_ML | NAD2L | NAD5L |
| 22L | 13TBM-12 | *M. laxa* | Unknown | Michigan (US) | MAT1-1 | Calmod7 | SDHA10 | TEF11 | Cyt_ML | NAD2L | NAD5L |
| 23L | 13LJIM-16 | *M. laxa* | Unknown | Michigan (US) | MAT1-1 | Calmod7 | SDHA10 | TEF11 | Cyt_ML | NAD2L | NAD5L |
| 24L | IV4-8 | *M. laxa* | Unknown | Michigan (US) | MAT1-2 | Calmod7 | SDHA10 | TEF11 | Cyt_ML | NAD2L | NAD5L |
| 25L | 13FSE20-3+ | *M. laxa* | Unknown | Michigan (US) | MAT1-1 | Calmod7 | SDHA10 | TEF11 | Cyt_ML | NAD2L | NAD5L |
| 26L | 13SHZM-2+ | *M. laxa* | Unknown | Michigan (US) | MAT1-1 | Calmod7 | SDHA10 | TEF11 | Cyt_ML | NAD2L | NAD5L |
| 27L | 13TBM-2 | *M. laxa* | Unknown | Michigan (US) | MAT1-1 | Calmod7 | SDHA10 | TEF11 | Cyt_ML | NAD2L | NAD5L |
| 28L | 13TBM-11 | *M. laxa* | Unknown | Michigan (US) | MAT1-2 | Calmod7 | SDHA10 | TEF11 | Cyt_ML | NAD2L | NAD5L |
| 29F | M.2.B.1.16.4 | *M. fructicola* | Blossom | Madeline (US), Musser Fruit Research Farm, Clemson, SC | MAT1-2 | Calmod3 | SDHA5 | TEF5 | Cyt_MF | NAD2 | NAD5 |
| 30F | M2.C1.6.16 | *M. fructicola* | Canker | Madeline (US), Musser Fruit Research Farm, Clemson, SC | MAT1-1 | Calmod2 | SDHA8 | TEF8 | Cyt_MF | NAD2 | NAD5 |
| 31F | M.3.C.3.16.6 | *M. fructicola* | Canker | Madeline (US), Musser Fruit Research Farm, Clemson, SC | MAT1-1 | Calmod2 | SDHA7 | TEF8 | Cyt_MF | NAD2 | NAD5 |
| 32F | M.4.C.7.16.3 | *M. fructicola* | Canker | Madeline (US), Musser Fruit Research Farm, Clemson, SC | MAT1-2 | Calmod2 | SDHA3 | TEF3 | Cyt_MF | NAD2 | NAD5 |
| 33F | M.4.C.7.16.11 | *M. fructicola* | Canker | Madeline (US), Musser Fruit Research Farm, Clemson, SC | MAT1-2 | Calmod2 | SDHA3 | TEF1 | Cyt_MF | NAD2 | NAD5 |
| 34F | M.4.B.7.16.3 | *M. fructicola* | Blossom | Madeline (US), Musser Fruit Research Farm, Clemson, SC | MAT1-2 | Calmod1 | SDHA1 | TEF3 | Cyt_MF | NAD2 | NAD5 |
| 35F | Z.1.C.6.16.7 | *M. fructicola* | Canker | Madeline (US), Sandy Springs, SC | MAT1-2 | Calmod2 | SDHA6 | TEF3 | Cyt_MF | NAD2 | NAD5 |
| 36F | Z.2.B.6.16.16 | *M. fructicola* | Blossom | Madeline (US), Sandy Springs, SC | MAT1-2 | - | - | TEF1 | Cyt_MF | NAD2 | NAD5 |
| 37F | Z2.C6.16.16 | *M. fructicola* | Canker | Madeline (US), Sandy Springs, SC | MAT1-1 | Calmod5 | - | TEF3 | Cyt_MF | NAD2 | NAD5 |
| 38F | Z.3.C.5.16.10 | *M. fructicola* | Canker | Madeline (US), Sandy Springs, SC | MAT1-1 | Calmod2 | - | TEF3 | Cyt_MF | NAD2 | NAD5 |
| 39F | Z.4.C.3.16.4 | *M. fructicola* | Canker | Madeline (US), Sandy Springs, SC | MAT1-1 | Calmod4 | SDHA1 | TEF10 | Cyt_MF | NAD2 | NAD5 |
| 40F | Z.4.B.3.16.7 | *M. fructicola* | Blossom | Madeline (US), Sandy Springs, SC | MAT1-2 | Calmod2 | - | TEF3 | Cyt_MF | NAD2 | NAD5 |
| CH1 | 2YTF5-1 | *M. fructicola* | Unknown | China (CH) | MAT1-2 | Calmod2 | SDHA2 | TEF1 | Cyt_MF | NAD2 | NAD5 |
| CH2 | 2THYF1-3 | *M. fructicola* | Unknown | China (CH) | MAT1-1 | Calmod2 | SDHA2 | TEF2 | Cyt_MF | NAD2 | NAD5 |
| CH3 | 2YTF2-2 | *M. fructicola* | Unknown | China (CH) | MAT1-1 | Calmod1 | SDHA2 | TEF3 | Cyt_MF | NAD2 | NAD5 |
| SHD3_TR | SHD-3 | *M. laxa* | Peach | Turkey (TR), Bursa/Chill Stor | MAT1-2 | Calmod7 | SDHA9 | TEF13 | Cyt_ML | NAD2L | NAD5L |
| 2B2A41_TR | 2B2-A4-1 | *M. laxa* | Peach | Turkey (TR), Çanakkale/Bayramiç | MAT1-1 | Calmod7 | SDHA10 | TEF11 | Cyt_ML | NAD2L | NAD5L |
| MMB3A4_TR | MM-B3-A4 | *M. laxa* | Plum | Turkey (TR), Mersin/Mezitli | MAT1-1 | Calmod7 | SDHA9 | TEF15 | Cyt_ML | NAD2L | NAD5L |
| MMB4A42_TR | MM-B4-A4-2 | *M. laxa* | Plum | Turkey (TR), Mersin/Mezitli | MAT1-1 | Calmod7 | SDHA9 | TEF11 | Cyt_ML | NAD2L | NAD5L |
| MMB5A3_TR | MM-B5-A3 | *M. laxa* | Plum | Turkey (TR), Mersin/Mezitli | MAT1-1 | Calmod7 | SDHA9 | TEF11 | Cyt_ML | NAD2L | NAD5L |
| MMB3A6_TR | MM-B3-A6 | *M. laxa* | Plum | Turkey (TR), Mersin/Mezitli | MAT1-1 | Calmod7 | SDHA9 | TEF11 | Cyt_ML | NAD2L | NAD5L |
| Yildirim2_TR | Yıldırım-2 | *M. laxa* | Peach | Turkey (TR), Bursa/Yıldırım | MAT1-2 | Calmod7 | SDHA9 | TEF12 | Cyt_ML | NAD2L | NAD5L |
| Yildirim1_TR | Yıldırım-1 | *M. laxa* | Peach | Turkey (TR), Bursa/Yıldırım | MAT1-2 | Calmod7 | SDHA9 | TEF12 | Cyt_ML | NAD2L | NAD5L |
| 2B1A5_TR | 2B1-A5 | *M. laxa* | Peach | Turkey (TR), Çanakkale/Bayramiç | MAT1-2 | Calmod7 | SDHA9 | TEF12 | Cyt_ML | NAD2L | NAD5L |
| TB1A42_TR | T-B1-A4-2 | *M. laxa* | Peach | Turkey (TR), İzmir/Belevi | MAT1-1 | Calmod7 | SDHA10 | TEF12 | Cyt_ML | NAD2L | NAD5L |
| MMB2A2_TR | MM-B2-A2 | *M. laxa* | Peach | Turkey (TR), Mersin/Mezitli | MAT1-1 | Calmod7 | SDHA9 | TEF14 | Cyt_ML | NAD2L | NAD5L |
| MMB4A4_TR | MM-B4-A4 | *M. laxa* | Peach | Turkey (TR), Mersin/Mezitli | MAT1-2 | Calmod7 | SDHA9 | TEF12 | Cyt_ML | NAD2L | NAD5L |
| MTB1A31_TR | MT-B1-A3-1 | *M. laxa* | Peach | Turkey (TR), Mersin/Tarsus | MAT1-2 | Calmod7 | SDHA9 | TEF14 | Cyt_ML | NAD2L | NAD5L |
| NiB3A2_TR | Ni-B3-A2 | *M. laxa* | Peach | Turkey (TR), Niğde | MAT1-2 | Calmod7 | SDHA9 | TEF14 | Cyt_ML | NAD2L | NAD5L |
| Bursa_Plum_TR | Bursa-Erik | *M. fructicola* | Plum | Turkey (TR), Bursa | MAT1-1 | Calmod1 | SDHA1 | TEF1 | Cyt_MF | NAD2 | NAD5 |
| BOB4A2_TR | BO-B4-A2 | *M. fructicola* | Peach | Turkey (TR), Bursa/Osmangazi | MAT1-2 | Calmod1 | SDHA2 | TEF1 | Cyt_MF | NAD2 | NAD5 |
| B1A1_TR | B1-A1 | *M. fructicola* | Peach | Turkey (TR), Çanakkale/Umurbey | MAT1-1 | - | SDHA1 | TEF1 | Cyt_MF | NAD2 | NAD5 |
| TB2A1_TR | T-B2-A1 | *M. fructicola* | Peach | Turkey (TR), İzmir/Belevi | MAT1-1 | Calmod2 | SDHA2 | TEF1 | Cyt_MF | NAD2i | NAD5i |
| MMB4A2_TR | MM-B4-A2 | *M. fructicola* | Peach | Turkey (TR), Mersin/Mezitli | MAT1-1 | Calmod1 | SDHA1 | TEF1 | Cyt_MF | NAD2 | NAD5 |
| NiB3A1_TR | Ni-B3-A1 | *M. fructicola* | Peach | Turkey (TR), Niğde | MAT1-2 | Calmod1 | SDHA1 | TEF1 | Cyt_MF | NAD2 | NAD5 |
| SCB5A2_TR | SC-B5-A2 | *M. fructicola* | Peach | Turkey (TR), Samsun/Çarşamba | MAT1-1 | Calmod1 | SDHA1 | TEF1 | Cyt_MF | NAD2 | NAD5 |
| SCB5A42_TR | SC-B5-A4-2 | *M. fructicola* | Peach | Turkey (TR), Samsun/Çarşamba | MAT1-1 | Calmod1 | SDHA1 | TEF1 | Cyt_MF | NAD2 | NAD5 |
| SCB1A1_TR | SC-B1-A1 | *M. fructicola* | Peach | Turkey (TR), Samsun/Çarşamba | MAT1-1 | Calmod1 | SDHA2 | TEF1 | Cyt_MF | NAD2 | NAD5 |
| 2B3A1_TR | 2B3-A1 | *M. fructicola* | Peach | Turkey (TR), Çanakkale/Bayramiç | MAT1-1 | Calmod2 | SDHA1 | TEF1 | Cyt_MF | NAD2i | NAD5i |
| B5A61_TR | B5-A6-1 | *M. fructicola* | Peach | Turkey (TR), Çanakkale/Umurbey | MAT1-1 | Calmod2 | SDHA1 | TEF1 | Cyt_MF | NAD2 | NAD5 |
| B5A2_TR | B5-A2 | *M. fructicola* | Peach | Turkey (TR), Çanakkale/Umurbey | MAT1-1 | Calmod1 | SDHA1 | TEF1 | Cyt_MF | NAD2 | NAD5 |
| SCB2A4_TR | SC-B2-A4 | *M. fructicola* | Peach | Turkey (TR), Samsun/Çarşamba | MAT1-2 | Calmod1 | SDHA1 | TEF1 | Cyt_MF | NAD2 | NAD5 |
| TB1A5_TR | T-B1-A5 | *M. fructicola* | Peach | Turkey (TR), İzmir/Belevi | MAT1-1 | Calmod2 | SDHA1 | TEF1 | Cyt_MF | NAD2 | NAD5 |
| TiB3A2_TR | Ti-B3-A2 | *M. fructicola* | Peach | Turkey (TR), İzmir/Tire | MAT1-2 | Calmod1 | SDHA1 | TEF1 | Cyt_MF | NAD2i | NAD5i |
| TiB3A32_TR | Ti-B3-A3-2 | *M. fructicola* | Peach | Turkey (TR), İzmir/Tire | MAT1-2 | Calmod1 | SDHA2 | TEF1 | Cyt_MF | NAD2i | NAD5 |
| B5A4_TR | B5-A4 | *M. fructicola* | Peach | Turkey (TR), Çanakkale/Umurbey | MAT1-1 | Calmod2 | SDHA1 | TEF1 | Cyt_MF | NAD2 | NAD5 |
| YK1_TR | Yol Kenarı 1 | *M. fructicola* | Peach | Turkey (TR), İzmir-Yol Kenarı | MAT1-2 | Calmod2 | SDHA2 | TEF1 | Cyt_MF | NAD2i | NAD5i |
| BGB1A4_TR | BG-B1-A4 | *M. fructicola* | Peach | Turkey (TR), Bursa/Gürsu | MAT1-1 | Calmod2 | SDHA2 | TEF1 | Cyt_MF | NAD2 | NAD5 |
| BGB1A17_TR | BG-B1-A17 | *M. fructicola* | Peach | Turkey (TR), Bursa/Gürsu | MAT1-2 | Calmod1 | SDHA1 | TEF1 | Cyt_MF | NAD2 | NAD5 |
|  |  |  |  |  |  |  |  |  |  |  | NAD5 |
| BGB1A13_TR | BG-B1-A13 | *M. fructicola* | Peach | Turkey (TR), Bursa/Gürsu | MAT1-2 | Calmod1 | SDHA1 | TEF1 | Cyt_MF | NAD2 | NAD5 |
| BGB1A15_TR | BG-B1-A15 | *M. fructicola* | Peach | Turkey (TR), Bursa/Gürsu | MAT1-1 | Calmod2 | SDHA2 | TEF1 | Cyt_MF | NAD2 | NAD5 |
| BGB1A6_TR | BG-B1-A6 | *M. fructicola* | Peach | Turkey (TR), Bursa/Gürsu | MAT1-2 | Calmod1 | SDHA2 | TEF2 | Cyt_MF | NAD2 | NAD5 |
| BGB3A1_TR | BG-B3-A1 | *M. fructicola* | Peach | Turkey (TR), Bursa/Gürsu | MAT1-2 | Calmod1 | SDHA2 | TEF1 | Cyt_MF | NAD2 | NAD5 |
| BGB4A3_TR | BG-B4-A3 | *M. fructicola* | Peach | Turkey (TR), Bursa/Gürsu | MAT1-2 | Calmod2 | SDHA2 | TEF1 | Cyt_MF | NAD2 | NAD5 |
| BOB1A3_TR | BO-B1-A3 | *M. fructicola* | Peach | Turkey (TR), Bursa/Osmangazi | MAT1-2 | Calmod1 | SDHA2 | TEF1 | Cyt_MF | NAD2 | NAD5 |
| BOB1A2_TR | BO-B1-A2 | *M. fructicola* | Peach | Turkey (TR), Bursa/Osmangazi | MAT1-2 | Calmod1 | SDHA2 | TEF1 | Cyt_MF | NAD2 | NAD5 |
| BOB3A1_TR | BO-B3-A1 | *M. fructicola* | Peach | Turkey (TR), Bursa/Osmangazi | MAT1-1 | Calmod1 | SDHA2 | TEF1 | Cyt_MF | NAD2 | NAD5 |
| SHD5_TR | SHD-5 | *M. fructicola* | Peach | Turkey (TR), Bursa/Chill Store | MAT1-2 | Calmod1 | SDHA2 | TEF1 | Cyt_MF | NAD2 | NAD5 |
| TY1_TR | ToplamaYeri1 | *M. fructicola* | Peach | Turkey (TR), Bursa/Gathering Point | MAT1-1 | Calmod1 | SDHA2 | TEF1 | Cyt_MF | NAD2 | NAD5 |
| 2B1A1_TR | 2B1-A1 | *M. laxa* | Peach | Turkey (TR), Çanakkale/Bayramiç | MAT1-1 | Calmod7 | SDHA10 | TEF11 | Cyt_ML | NAD2L | NAD5L |
| 2B1A21_TR | 2B1-A2-1 | *M. laxa* | Peach | Turkey (TR), Çanakkale/Bayramiç | MAT1-1 | Calmod7 | SDHA10 | TEF11 | Cyt_ML | NAD2L | NAD5L |
| 2B1A22_TR | 2B1-A2-2 | *M. laxa* | Peach | Turkey (TR), Çanakkale/Bayramiç | MAT1-1 | Calmod7 | SDHA10 | TEF11 | Cyt_ML | NAD2L | NAD5L |
| 2B2A1_TR | 2B2-A1 | *M. laxa* | Peach | Turkey (TR), Çanakkale/Bayramiç | MAT1-1 | Calmod7 | SDHA10 | TEF11 | Cyt_ML | NAD2L | NAD5L |
| 2B3A3_TR | 2B3-A3 | *M.laxa* | Peach | Turkey (TR), Çanakkale/Bayramiç | - | Calmod7 | SDHA10 | TEF13 | Cyt_MF | NAD2 | NAD5 |
| B5A72_TR | B5-A7-2 | *M. fructicola* | Peach | Turkey (TR), Çanakkale/Umurbey | MAT1-2 | Calmod2 | SDHA1 | TEF1 | Cyt_MF | NAD2 | NAD5 |
| B5A71_TR | B5-A7-1 | *M. fructicola* | Peach | Turkey (TR), Çanakkale/Umurbey | MAT1-1 | Calmod1 | SDHA1 | TEF1 | Cyt_MF | NAD2 | NAD5 |
| B5A62_TR | B5-A6-2 | *M. fructicola* | Peach | Turkey (TR), Çanakkale/Umurbey | - | - | SDHA1 | TEF1 | Cyt_MF | NAD2 | NAD5 |
| B1A3_TR | B1-A3 | *M. fructicola* | Peach | Turkey (TR), Çanakkale/Umurbey | MAT1-2 | Calmod1 | SDHA1 | TEF1 | Cyt_MF | NAD2 | NAD5 |
| YK2_TR | Yol Kenarı-2 | *M. fructicola* | Peach | Turkey (TR), İzmir/Yol Kenarı | MAT1-2 | Calmod1 | SDHA1 | TEF1 | Cyt_MF | NAD2i | NAD5 |
| TB3A4_TR | T-B3-A4 | *M. fructicola* | Peach | Turkey (TR), İzmir/Belevi | MAT1-1 | Calmod1 | SDHA2 | TEF1 | Cyt_MF | NAD2 | NAD5 |
| TB1A6_TR | T-B1-A6 | *M. laxa* | Peach | Turkey (TR), İzmir/Belevi | MAT1-1 | Cal3 | SDHA9 | TEF1 | Cyt_ML | NAD2L | NAD5 |
| TB4A23_TR | T-B4-A2-3 | *M. fructicola* | Peach | Turkey (TR), İzmir/Belevi | MAT1-1 | Calmod1 | SDHA2 | - | Cyt_MF | NAD2 | NAD5 |
| SB1A1_TR | SB1-A1 | *M. fructicola* | Peach | Turkey (TR), İzmir/Selçuk | MAT1-1 | Calmod2 | SDHA2 | TEF1 | Cyt_MF | NAD2i | NAD5i |
| TiB4A22_TR | Ti-B4-A2-2 | *M. fructicola* | Peach | Turkey (TR), İzmir/Tire | MAT1-2 | Calmod2 | SDHA2 | TEF1 | Cyt_MF | NAD2 | NAD5 |
| TiB6A1_TR | Ti-B6-A1 | *M. fructicola* | Peach | Turkey (TR), İzmir/Tire | MAT1-1 | Calmod1 | SDHA1 | TEF1 | Cyt_MF | NAD2 | NAD5 |
| TiB5A1_TR | Ti-B5-A1 | *M. fructicola* | Peach | Turkey (TR), İzmir/Tire | MAT1-1 | Calmod2 | SDHA2 | TEF1 | Cyt_MF | NAD2i | NAD5i |
| MMB4A10_TR | MM-B4-A10 | *M. laxa* | Peach | Turkey (TR), Mersin/Mezitli | MAT1-2 | Calmod7 | SDHA10 | TEF11 | Cyt_ML | NAD2L | NAD5L |
| MMB4A3_TR | MM-B4-A3 | *M. laxa* | Peach | Turkey (TR), Mersin/Mezitli | MAT1-1 | Calmod7 | SDHA10 | TEF11 | Cyt_ML | NAD2L | NAD5L |
| MMB4A1_TR | MM-B4-A1 | *M. laxa* | Peach | Turkey (TR), Mersin/Mezitli | MAT1-2 | Calmod7 | SDHA10 | TEF11 | Cyt_ML | NAD2L | NAD5L |
| MMB4A5_TR | MM-B4-A5 | *M. laxa* | Peach | Turkey (TR), Mersin/Mezitli | MAT1-2 | Calmod7 | SDHA10 | TEF11 | Cyt_ML | NAD2L | NAD5L |
| NiB4A1_TR | Ni-B4-A1 | *M. laxa* | Peach | Turkey (TR), Niğde | MAT1-2 | Calmod7 | SDHA10 | TEF11 | Cyt_ML | NAD2L | NAD5L |
| SCB3A2_TR | SC-B3-A2 | *M. fructicola* | Peach | Turkey (TR), Samsun/Çarşamba | MAT1-1 | Calmod1 | SDHA2 | TEF1 | Cyt_MF | NAD2 | NAD5 |
| SCB4A82_TR | SC-B4-A8-2 | *M. fructicola* | Peach | Turkey (TR), Samsun/Çarşamba | MAT1-2 | Calmod1 | SDHA1 | TEF1 | Cyt_MF | NAD2 | NAD5 |
| SCB5A7_TR | SC-B5-A7-1 | *M. fructicola* | Peach | Turkey (TR), Samsun/Çarşamba | MAT1-2 | Calmod1 | SDHA1 | TEF1 | Cyt_MF | NAD2 | NAD5 |
| SCB4A81_TR | SC-B4-A8-1 | *M. fructicola* | Peach | Turkey (TR), Samsun/Çarşamba | MAT1-2 | Calmod1 | SDHA1 | TEF1 | Cyt_MF | NAD2 | NAD5 |
| SCB3A1_TR | SC-B3-A1 | *M. fructicola* | Peach | Turkey (TR), Samsun/Çarşamba | MAT1-1 | Calmod1 | SDHA2 | TEF1 | Cyt_MF | NAD2 | NAD5 |
| SCB5A5_TR | SC-B5-A5 | *M. fructicola* | Peach | Turkey (TR), Samsun/Çarşamba | MAT1-2 | Calmod1 | SDHA2 | TEF1 | Cyt_MF | NAD2 | NAD5 |
| SCB2A2_TR | SC-B2-A2 | *M. fructicola* | Peach | Turkey (TR), Samsun/Çarşamba | MAT1-1 | Calmod1 | SDHA1 | TEF1 | Cyt_MF | NAD2 | NAD5 |
| SCB5A41_TR | SC-B5-A4-1 | *M. fructicola* | Peach | Turkey (TR), Samsun/Çarşamba | MAT1-2 | Calmod1 | SDHA2 | TEF1 | Cyt_MF | NAD2 | NAD5 |
| SCB4A1_TR | SC-B4-A1 | *M. fructicola* | Peach | Turkey (TR), Samsun/Çarşamba | MAT1-1 | Calmod1 | SDHA1 | TEF1 | Cyt_MF | NAD2 | NAD5i |
