# Supplementary material for: Phylogenetic divergences in brown rot fungal pathogens of Monilinia species from a worldwide collection: inferences based on the nuclear versus mitochondrial genes
Source: BMC Ecol Evol. 2022 Oct 21;22:119. doi: 10.1186/s12862-022-02079-6 (PMC9585774; doi:10.1186/s12862-022-02079-6)
Supplement: Supplementary file 10 — Additional file 10: Table S2. Table of every primer used in this study. [file 12862_2022_2079_MOESM10_ESM.docx]

**Supplementary Table 2** Table of every primer used in this study

| **Gene Region** | **Primers** | **Primer Sequences** | **Reference** |
| --- | --- | --- | --- |
| Cytochrome-b | Moncytb-F | TGGGTAATCCGCAGCCAAG | This study |
|  | Moncytb-R | ATTCGGCGCTTTCCACAGA | This study |
| NAD5 | MonNAD5-FW1 | TTGGTGCTATGGCTAAAAGTTCT | This study |
|  | MonNAD5-FW2 | ATCCGGGTTTACAGATGCTG | This study |
|  | MonNAD5-REV | ACCTACTGCTATTACCATCATACCT | This study |
| NAD2 | MonNAD2-FW | ACAAGCGGTGGTTTGACCTA | This study |
|  | MonNAD2-REV1 | AAACTTAAGGCTAAGACAGGGT | This study |
|  | MonNAD2-REV2 | TCGCCTCGTGTCAATTACCG | This study |
| TEF1  (for *M. laxa*) | EF1-728F | CATCGAGAAGTTCGAGAAGG | Carbone & Kohn, 1999 |
|  | EF1-986R | TACTTGAAGGAACCCTTACC | Carbone & Kohn, 1999 |
| TEF1  (for *M. fructicola*) | MFTEF1-F | TAAGCCATATCCAGCCTGCA | This study |
|  | MFTEF1-R | GGTGTCTCGAACTTCCAGAG | This study |
| SDHA1 | MFSDHA1-F | GGACAGCCTTGCACTCATTC | This study |
|  | MFSDHA1-R | CGAGCGTCTTCCAGGTATCT | This study |
| Calmodulin | MFCalmod-F | TGCCGTACCTGGGAAATCAA | This study |
|  | MFCalmod-R | ACACTTGGCAGGGCATATCT | This study |
