# Supplementary material for: Phylogenetic divergences in brown rot fungal pathogens of Monilinia species from a worldwide collection: inferences based on the nuclear versus mitochondrial genes
Source: BMC Ecol Evol. 2022 Oct 21;22:119. doi: 10.1186/s12862-022-02079-6 (PMC9585774; doi:10.1186/s12862-022-02079-6)
Supplement: Supplementary file 11 — Additional file 11: Table S3. Table of each regions substitution rates. Outer left column shows used software, second column indicates algorithms used. [file 12862_2022_2079_MOESM11_ESM.docx]

**Supplementary Table 3** Table of each regions substitution rates. Outer left column shows used software, second column indicates algorithms used.

| Software | Algorithm | *Calmodulin* | *SDHA* | *NAD2* | *NAD5* | *Cytb* | *TEF1* |
| --- | --- | --- | --- | --- | --- | --- | --- |
| jModelTest2 | AIC | TVM | TIM2ef | TPM2uf | HKY | TPM3uf | TPM2 |
|  | AICc | K80 | TIM2ef | HKY | HKY | F81 | K80 |
|  | BIC | K80 | TrNef | HKY | F81 | F81 | K80 |
|  | DT | K80 | TrNef | HKY | F81 | JC | TPM2 |
| mrmodeltest2 | HLRT | K80 | SYM | HKY | F81 | F81 | F81 |
|  | AIC | GTR | SYM | HKY | HKY | GTR | HKY |
